# Gene expression profiling of rat spermatogonia and Sertoli cells reveals signaling pathways from stem cells to niche and testicular cancer cells to surrounding stroma

**DOI:** 10.1186/1471-2164-12-29

**Published:** 2011-01-13

**Authors:** Stephan Ryser, Dominique Glauser, Michelle Vigier, Yong Qiang Zhang, Philippe Tachini, Werner Schlegel, Philippe Durand, Irmgard Irminger-Finger

**Affiliations:** 1 Molecular Gynecology and Obstetrics Laboratory, Geneva University Hospitals, Geneva, Switzerland; 2 Fondation pour Recherches Médicales, Geneva, Switzerland; 3 INSERM, UMR 418; INRA, UMR 1245, Debrousse Hospital, Lyon, France; 4 EDEL Therapeutics, Lausanne, Switzerland; 5 Institut de Génomique Fonctionnelle de Lyon, Université de Lyon; Université Lyon 1; CNRS; INRA; Ecole Normale Supérieure de Lyon, Lyon, France; 6 Lausanne University Hospital, Lausanne, Switzerland

## Abstract

**Background:**

Stem cells and their niches are studied in many systems, but mammalian germ stem cells (GSC) and their niches are still poorly understood. In rat testis, spermatogonia and undifferentiated Sertoli cells proliferate before puberty, but at puberty most spermatogonia enter spermatogenesis, and Sertoli cells differentiate to support this program. Thus, pre-pubertal spermatogonia might possess GSC potential and pre-pubertal Sertoli cells niche functions. We hypothesized that the different stem cell pools at pre-puberty and maturity provide a model for the identification of stem cell and niche-specific genes. We compared the transcript profiles of spermatogonia and Sertoli cells from pre-pubertal and pubertal rats and examined how these related to genes expressed in testicular cancers, which might originate from inappropriate communication between GSCs and Sertoli cells.

**Results:**

The pre-pubertal spermatogonia-specific gene set comprised known stem cell and spermatogonial stem cell (SSC) markers. Similarly, the pre-pubertal Sertoli cell-specific gene set comprised known niche gene transcripts. A large fraction of these specifically enriched transcripts encoded trans-membrane, extra-cellular, and secreted proteins highlighting stem cell to niche communication. Comparing selective gene sets established in this study with published gene expression data of testicular cancers and their stroma, we identified sets expressed genes shared between testicular tumors and pre-pubertal spermatogonia, and tumor stroma and pre-pubertal Sertoli cells with statistic significance.

**Conclusions:**

Our data suggest that SSC and their niche specifically express complementary factors for cell communication and that the same factors might be implicated in the communication between tumor cells and their micro-enviroment in testicular cancer.

## Background

The balance between self-renewal and differentiation of stem cells is tightly regulated during embryonic development of higher eukaryotes. This control is defined by intrinsic genetic programs within the stem cells and by extracellular cues from the surrounding cells. Stem cells are surrounded by a specialized microenvironment termed "niche," which promotes self-renewal and maintenance of stem cells in their undifferentiated state. Niche cells produce extracellular components surrounding the stem cells, as well as factors of cell-cell contact, and signaling molecules related to stem cell support functions [1-3]. Much of our understanding of the molecular features of the stem cell niches comes from the work on *C. elegans *and *Drosophila*. In these species, molecular mechanisms and genes involved in maintaining germline stem cells and their niche have been characterized. In contrast, little is known about the less well defined mammalian germ stem cells and the somatic support cells that form the niche [2,4].

Spermatogenesis is a highly organized process which consists of three distinct phases during adulthood: mitosis, meiosis and spermiogenesis. In rodents, meiosis and spermiogenesis are only initiated at puberty. Mitotic germ cells are spermatogonia (Spga) that originate from primordial germ cells (PGCs) in the embryo. In the adult testis, Spga are localized to the basement membrane of the seminiferous tubule, and Spga differentiation during meiosis are taking place along a gradient towards the lumen of the seminiferous tubule [5-9]. Spga can be sub-divided into two morphological groups: undifferentiated Spga (type A_single_, A_paired, _and A_aligned_) and the differentiated Spga (type A_1_-A_4_, Intermediate, and type B Spga). Type A_single _Spga are defined as spermatogonial stem cells (SSCs) and are localized most proximal to the basement membrane of the seminiferous tubule [5-9]. Spga A_paired _and A_aligned _are already committed to differentiation, but maintain similar morphological and cellular properties as Spga A_single_, and are called undifferentiated spermatogonia [5-9]. Several groups have shown that undifferentiated Spga of the first wave of spermatogenesis comprise a large fraction of cells with stem cell characteristics and self renewal potential [10-13]. Thus, Spga in pre-pubertal testis are highly enriched in cells with stem cell potential.

Sertoli cells are the supporting somatic cells essential for the development of male germ cells of all stages, including Spga. Before puberty Sertoli cells provide niche functions for Spga, stimulating their proliferation and self-renewal. At puberty, mature Sertoli cells acquire new functions to support the onset of meiosis. Tight junctions are created between the Sertoli cells to separate the niche of mitotic Spga from the niche required for meiotic cells, the latter niche producing hormones and paracrine factors that drive sperm diffentiation [14,15].

Before puberty, immature Sertoli cells provide proliferation and differentiation signals for Spga. Immature Sertoli cells proliferate in parallel to Spga until the seminiferous epithelium reaches its final size. At each division of pre-pubertal Sertoli cells, the daughter cells generate specialized micro-domains to sustain the amplification of the mitotic Spga. Sertoli cells thus maintain the potential of a stem cell niche for dividing SSCs. This was demonstrated by transplantation studies, which showed that pre-pubertal rodents support higher levels of donor germ cell engraftment than adult animals [16-18]. Apparently, an increased number of niche cells in recipient pups, which had endogenous germ cells removed or compromised by busulfan treatment, favors the engraftment of donor stem cells in animals, [19]. Reciprocally, an increase of engraftment was observed in recipient adult busulfan-treated mice, when transplanted germ cells from prepubertal donors (4-5 dpp) were compared to Spga from pubertal animals (28 dpp). These experiments suggest that pre-pubertal testis contain a large proportion of Spga with stem cell potential [20] and of Sertoli cells that fulfill niche functions.

Based on these observations, we reasoned that comparing the gene expression profiles of Spga from pre-pubertal and pubertal animals would lead to the identification of stemness-specific genes. Similarly, comparing the expression profiles of the supporting Sertoli cells in pre-pubertal and pubertal animals should lead to identification of niche-specific gene expression. The transcriptomes of mitotic germ cells or Sertoli cells have been analyzed previously in isolated preparations of adult testis [21-27], but their comparison at different stages of development has not been addressed.

Spga, and in particular GSC, are believed to be the origin of the most frequent types of testicular cancers: seminomas and non-seminomas. Indeed, expression of embryonic stem cell markers was found in human seminomas, non-seminomas, and the precursor lesion of testicular germ cell cancer [28-30]. We therefore hypothesized that the analysis of the expression profile of SSCs and their niche should lead to identification of factors that are also important in signaling between testicular cancers and their tumor micro-environment.

In this study, we compared expression profiles of Spga with Sertoli cells purified from pre-pubertal and pubertal rats and established gene lists that characterized the stem cell and niche potential of pre-pubertal Spga and Sertoli cells, respectively. Secondly, we compared the SSC-specific genes and Sertoli cell (niche cell)-specific genes to genes upregulated in testicular cancers and genes specifically expressed in tumor stroma. Functional data mining, and quantitative PCR performed for a selection of candidate genes, highlighted the coinciding upregulated expression of functionally interacting products in Spga and Sertoli cells, suggesting that certain cell adhesion proteins and secreted factors interacting with their receptors were specifically involved in the essential interaction between SSCs and their niche, the Sertoli cells. Published gene expression profiles of testis cancer showed a highly significant overlap with gene sets over-expressed in pre-pubertal Spga and Sertoli cells underlining the relevance of the SSC to niche communication in the development of testis cancer.

## Results

### Purification of SSC-enriched Spga and Sertoli cells

**Figure 1:**
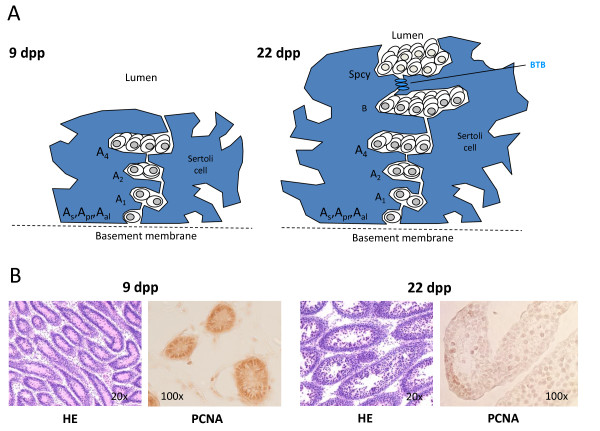
**Proliferation and differentiation of germ cells and Sertoli cells of young rats at two different stages of development**. ***(A) ***Schematic presentation of differentiation stages of germ cells (Spermatogonia A_s_, A_pr_, A_al_, A_1_, A_2_, A_4_, and B, and Spermatocytes (Spcy)) and Sertoli cells (blue shaded area) which surround the germ cells of pre-pubertal (9 dpp) and pubertal (22 dpp) rats. Blood-testis barrier (BTB) formed by Seroli cells at 9 dpp is indicated. ***(B) ***Sections of seminiferous tubules were stained with hematoxylin (HE) and antibodies against proliferating cell nuclear antigen (PCNA) to confirm proliferation of all spermatogonia at 9 dpp, but few at 22 dpp.

The maturation of the testis during puberty involves a concerted change in the populations of germline cells and of their supporting Sertoli cells (Figure 1A). Immature rat Sertoli cells at 9 dpp grow rapidly and divide, stop dividing at 15 dpp, and are mature at 22 dpp having established the blood-testis barrier by forming tight junctions among themselves. The germ cell population at 9 dpp contains type A Spga, which show rapid proliferation and self-renewal. At 22 dpp, the germ cell fraction that we isolated still contains Spga type A, but also intermediary, type B, and preleptotene spermatocytes [31-34].

We purified Spga and Sertoli cells from testis of pre-pubertal rats at 9 dpp and pubertal rats at 22 dpp. To demonstrate the proliferative stages of both cell types at 9 dpp, we stained sections from rat testis at 9 dpp and 22 dpp with the mitotic marker Proliferative Cell Nuclear Antigen (PCNA). PCNA was highly expressed at 9 dpp; PCNA staining was decreased at 22 dpp (Figure 1B), consistent with a lower frequency of mitosis due to the shift of the germ cell population towards differentiated spermatocytes and the maturation of Sertoli cells. These concerted changes present an opportunity to study differential gene expression of SSC and their niche by cross-comparison of purified Spga and Sertoli cells at two crucial stages of testis development.

### Identification of differentially expressed transcripts in Spga and Sertoli cells of pre-pubertal and pubertal rats

To identify genes that are specific for SSCs and their niche, we compared gene expression profiles of spermatogonia and Sertoli cells prepared from testis of pre-pubertal and pubertal rats at 9 and 22 dpp, respectively. We established 4 expression profiles, namely for spermatogonia (G) and Sertoli (S) cells, of post-natal day 9 or 22, termed G9, S9, G22 and S22. Each profile was based on three sets of separately purified spermatogonia or Sertoli cells, which were used for mRNA preparation and microarray hybridization.

**Figure 2:**
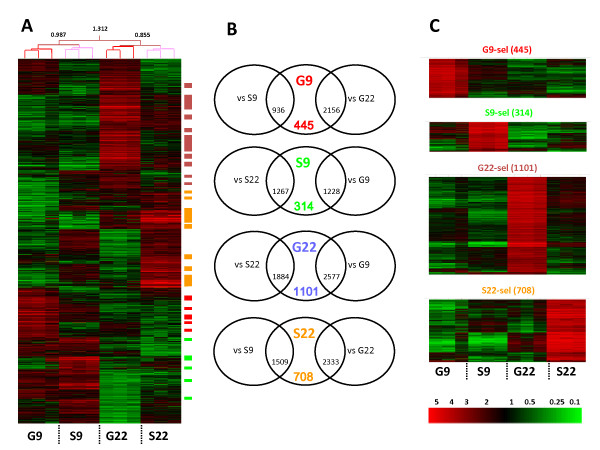
**Expression profiles of Spga and Sertoli cells from pre-pubertal and pubertal rats**. ***(A) ***Triplicate profiles of the 6908 genes which were differentially expressed in the 4 types of cells (germ cells at 9 dpp and 22 dpp {G9 and G22 respectively} and Sertoli cells at 9 dpp and 22 dpp respectively) were hierarchically clustered and are presented as a heat-map. Hierarchy and deviance are shown on top of the heat-map. Expression level of each transcript is indicated in the color code bar, red for high and green for low expression. ***(B) ***Venn diagrams showing genes which are at least twofold over-expressed in pair wise comparison of mean expression levels between either the same cell type (e.g. G9 vs G22) or the same developmental stage (e.g. G9 vs. S9). Overlap defines 4 "selective" gene sets: G9-sel (red); S9-sel (green); G22-sel (blue); S22-sel (orange). Genes of these 4 sets are marked by small colored bars on the right of the heat-map in panel A. (C) Hierarchical re-clustering of the 4 "selective" gene sets. Gene tree clustering parameters: Pearson Correlation, average linkage algorithm.

Of 31099 genes on the microarray, 6908 transcripts were expressed at a level above the threshold defining differential expression (see Methods) and at least two fold up-regulated over the other groups. Hierarchical clustering of differentially expressed transcripts of the four cell preparations showed that G9, S9, G22, and S22 formed distinct groups (Figure 2A). The clustering tree showed that the differences in expression patterns were less pronounced between the cell types of the same developmental stage (between G9 and S9 or between G22 and S22), than between different developmental stages of one cell type (between G9 and G22, and S9 and S22). Specifically, Sertoli cells and Spga shared a substantial set of similarly expressed transcripts at 9 dpp. Such overlapping gene expression pattern is consistent with the mitotic program that is common to both cell types.

We then performed pair-wise comparison of two-fold enriched transcripts of the G9, G22, S9, and S22 gene sets. As shown in Venn diagrams (Figure 2B), the cross-section of the gene lists of the pair-wise comparison (e.g. gene list of G9 versus G22 and versus S9) revealed 445, 314, 1101, and 708 transcripts that were selectively enriched in G9 and S9, G22, and S22, respectively. These four "selective" gene sets were termed G9-sel, S9-sel, G22-sel, S22-sel. As shown in Figure 2C, hierarchical re-clustering of each "selective" gene set revealed specifically enriched (minimally 2-fold) transcripts in each cell population and no overlap with the three other cell populations. Thus, all four cell fractions have their own unique, divergent gene expression profiles.

### Functional data mining

**Figure 3:**
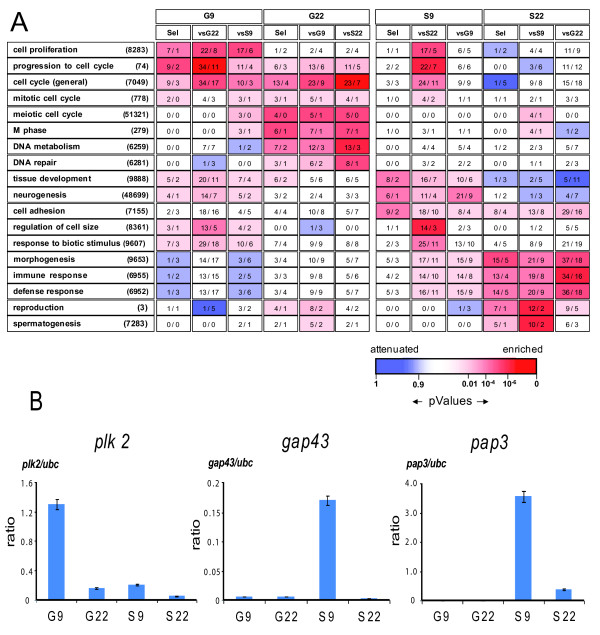
**Gene ontology (GO) category enrichment in the differentially up-regulated gene clusters present in pre-pubertal and pubertal germ cells and Sertoli cells**. ***(A) ***Gene ontology categories for transcripts enriched or attenuated in G9 and S9 cells. Number of transcripts associated with a specific GO category and enriched in a cluster are given within rectangles as observed and as expected values. For each GO category, P values are shown by a gradual color code bar. Red indicates over-representation of transcripts in a GO category whereas blue specifies under-representation. The gene sets up-regulated in one condition (as defined in Figure 2B) were analyzed here for GO enrichment and ordered according to the peak expression found in pre-pubertal (G9) and pubertal (G22) germ cells and in Sertoli cells (S9 and S22). Only the most enriched GO categories are displayed here for the four cell populations. ***(B) ***Validation of Spga and Sertoli cell specific gene expression at pre-puberty by RT-PCR. Expression of Polo-like kinase 2 (plk2), growth associated protein 43 (gap43) and pancreatitis associated protein 3 (pap3) in prepubertal (9 dpp) and pubertal (22 dpp) spermatogonia and Sertoli cells (G9, G22. S9, S22, respectively) was quantified by real time RT-PCR. Values normalized to the housekeeping genes ubc are shown; the same pattern was observed when normalizing with the expression of ps9, the other housekeeping gene (not shown). Shown are the mean values (± SEM) from the 3 distinct cell preparations, for which genome-wide analysis by hybridization to a high density array is shown in Figure 2.

To evaluate whether the differentially expressed genes reflected specific biological functions, we assigned them to gene ontology (GO) categories. This analysis revealed the functional networks involved in the onset of spermatogenesis (Figure 3A). The G9 set contained genes involved in "cell proliferation", "progression through cell cycle" or "cell cycle". Transcripts of the same GO categories were enriched in the S9 set when compared to the S22 set. These results are consistent with the fact that both Spga A and Sertoli cells are proliferating in pre-pubertal rat testis [31-35]. As expected, gene transcripts involved in "meiotic cell cycle", "M phase", "DNA metabolism", and "DNA repair", were enriched in the G22 sets. These gene sets overlapped significantly with the mitotic and meiotic germ cell gene clusters defined by Chalmel et al. [21] (Additional file 1, Table S1).

Genes that were most upregulated in the S9 set comprised a significantly elevated number of genes of the GO categories "cell adhesion", "neurogenesis", "tissue development", "morphogenesis", and "regulation of cell size" (Figure 3A). Mature Sertoli cells in the adult have to support a larger number of germ cells than immature Sertoli cells in pre-pubertal rats, and accompany their differentiation [36]. Presumably, the expression of genes associated with "morphogenesis" and "cell size" in S22 reflects commitment to maturation of Sertoli cells and to the onset of spermiogenesis. "Tissue development" and "neurogenesis" genes were highly expressed in S9, but were under-expressed in S22. This suggested that during the first wave of spermatogenesis at 22 dpp, the Sertoli cells are fully mature. Further, we observed a significant enrichment of the GO "immune or defense response", "response to biotic stimulus", "reproduction", and "spermatogenesis" in S22. The up-regulated expression of genes of the immune system in S22 presumably reflects the physiological process that leads to immuno-tolerance in the testis [37]. Thus, the gene categories up-regulated in the different gene sets are consistent with the functions attributed to Spga and Sertoli cells. In the "selective" gene sets the enrichment of these GO categories was similar despite the smaller list of transcripts.

To identify the most differentially expressed genes, we sorted the "selective" gene sets by their fold change of mean expression and classified them in 14 functional categories. Listing only genes with a minimum 4 fold variation of mean expression, we defined 176 genes for G9 and 170 for S9 (Table 1). Among the "cell cycle" genes upregulated in G9 were Gadd45gamma (Gadd45g), Myc, and Polo-like-kinase 2 (Plk2). S9 cells expressed significantly more inhibitors of cell cycle, such as Cdkn2a (ARF/INK4) and Mtsg1. The most up-regulated genes in the GO category "neurogenesis" were in S9, namely Growth Associated Protein 43 (GAP43) and Prepronociceptin (Pnoc). The most prominent gene transcripts within the "cell adhesion, ligand, ECM" category, differentially enriched in S9 were pap/REG3, thrombomodulin (Thpd), and Ncam1. A complete description of the genes listed in Table 1 is shown in Additional file 1, Tables S2 and S3.

**Table 1:** The most up-regulated genes found in type A spermatogonia and Sertoli cells at 9dpp.

	Mitotic Spermatogonia type A at 9dpp	Immature Sertoli cells at 9dpp
Cell adhesion, ligand, ECM	(12) *Alcam, Bdnf, Ctgf, Cyr61, Daf1, Hbegf*,	(19) Asam, Boc*, Cdh11, Cdh22, Col4a1*, Itgav*, Mdk, Pdgfc, Pvr, Tpbg, Vasn*, Col4a2*, Cthrc1, Cxcl13, Fbn1, Gpc1, Madcam1, Ncam1, Npy, Pap, Pap3, Sfrp1, Spp1, Thbd, Torid
Cytoskeleton	(5) Actn1, Coro1a, Mig12, Nef3, Sprr1*	(8) Dbn1, Mybpc3*, Nes, Pdlim2, Pdlim3, Tmod1, Trim2*, Tubb2b*
Cell cycle, Apoptosis	(6) Gadd45a, Gadd45b*, Gadd45g*, Myc, Plk2, Tpd52l1*,	(3) *Cdkn2a, Cdkn2b, Mtsg1*
Intracellular membranes, Trafficking	(3) Mal2, Scrn1, Snx7*	(2) Ech1, Tmem98*
Metabolism	(10) Akr1c18, Gls, Gpx1, Lpl, Prkaa1, Qpct*, Rfk*, Sms*,Txnip, Ugcg	(12) Ddah2, Cbr3*, Cox6a2, Cp, Crym, Dio3, Gpx7*, Gsta2, Idh2, Mgst2*, Pygl, SelM*
RNA Splicing, translation	(2) *Rpl13, Rpl37*	(1) *Hspb1*
Receptors, channels, transporters	(11) Cd7*, Edn1, F2r, Gnai1, Lgr4, Nritp, P2ry1, Slc4a4, Slc6a15, Slc7a3, Slc16a6	(11) Gpr83*, Kdr, Ntrk1, Scn3b, Scn4b, Slc15a2, Slc26a3, Smstr28*, Sntg2*, Tacstd2, Trfr2*
Signal transduction	(13) Anxa3, Arhgap18*, Arhgap21*, Dusp1, Hipk3, Ppp1r14c, Ptpre, Rnd3, Sdpr, Sgk, Tm4sf12, Trib2*, Trib3	(9) Arhgef4*, Cpne8, Ptpns1, Ltbp2, Rerg*, Rrad, S100a3, S100a5*, Smoc2*
Transcription, chromatin	(11) Anp32a, Atf3, Ets1, Fosl1, Hes1, Mycn, Nap1l3, Parp8*, Rb1, Tle4, Zfp469*	(9) Giot1, Lmcd1*, Nr4a1, Nupr1, Rgc32, Sox18*, Tead2, Znf292, Znf704*
Angiogenesis, Immune response	(3) *F3, Tmem23, Vegfc*	(1) *Thbd*
Development	(7) *Bmp2, Bmp4, Id2, Inha, Inhbb, Kitl, Nog*	(3) *Bmf, Inhba, Mgp*
Neurogenesis	-	(9) Apoe, Dscr1l1, Efna1, Gap43, Ntf5, Penk-rs, Plxnb1*, Pnoc, Rogdi*
Proteolysis, peptidolysis	(3) *Gzmc, Lcn7, Prss23*	(9) Adam10, Adam33*, Htra3*, Masp1, Plau, Plat, Plxnc1*, Tessp6, Ube2c
Others	(90) *37 predicted EST, 53 unknown EST*	(74) *33 predicted EST, 41 unknown EST*
**Total**	**(176)**	**(170)**

To confirm the specific differential expression of genes by other means, we quantified a small set of relevant gene transcripts by RT-PCR in total RNA extracted from the same cell preparations by the same procedures as for microarray hybridization. Figure 3B shows three typical examples: *plk2 *is specifically expressed in G9 cells, and expression is nearly absent in the three other cell preparations. The *gap43 *and *pap3 *genes are expressed specifically in S9 cells, and expression is completely absent in the other cell types. This supports the notion that *gap43 *and *pap3 *have stage-specific functions in pre-pubertal Sertoli cells and that *plk2 *is relevant for pre-pubertal spermatogonia.

### Identification of SSC marker genes

**Figure 4:**
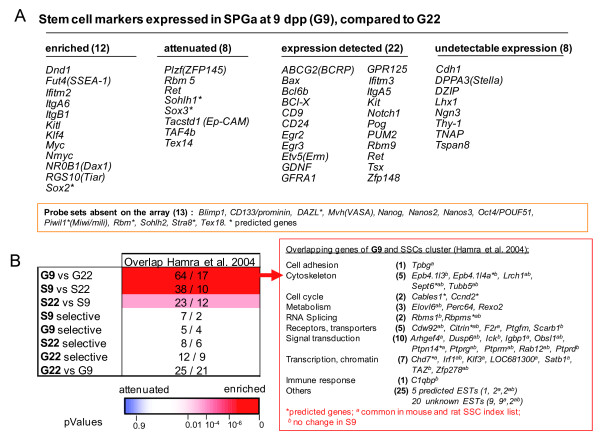
**Identification of SSC genes and markers in pre-pubertal type A spermatogonia**. ***(A) ***Rat Affymetrix probe set IDs for ES, PGC, gonocyte and SSC markers were selected and analyzed for expression in the Spga cell fractions at 9 and 22 dpp. Genes up-regulated or down-regulated at least 2 fold in type A Spga (G9) compared to mitotic germ cells at 22 dpp (G22) are shown in the first two columns. Genes with a detectable or undetectable expression in the two last columns (defined here as the number of Affymetrix Present/Absent in the replicates after MAS 5.0 analysis). ***(B) ***The 255 rat SSC-specific transcripts identified in a microarray analysis by Hamra et al. [23] were compared to each respective gene cluster defined in Figure 2B. *Left*: Shown are the number of transcripts in each cluster (e.g. G9 vs G22) which overlapped with the transcript list by Hamra et al. [23] as compared to the number of randomly expected transcripts (64/17; i.e. 64 observed versus 17 expected in a random distribution). The gradual color code bar shows over-representation (red) and under-representation (blue) of SSC specific gene transcripts described by Hamra et al. [23] in the various clusters defined in this study (Figure 2B). Significance of overlap (p Values) was calculated using a Gaussian hypergeometric test (Fischer exact probability test). *Right*: Gene list and functional classification of the genes up-regulated in mitotic Spga type A at 9 dpp overlapping with the rat SSC cluster predicted by Hamra et al [23].

Pre-pubertal Spga comprise all subtypes of undifferentiated and differentiated type A Spga, but their respective abundances are not clear [19,38,39]. To evaluate stem cell characteristics of pre-pubertal Spga, we first established a list of 63 genes, composed of selected markers of embryonic stem cells, primordial germ cells (PGCs), gonocytes, or Spga from published data (Figure 4A). Of these 63 genes, 50 were represented on the RAE230 2.0 gene chip used in this study. Most of these markers were present in the G9 and G9-sel gene sets. Not included in differentially expressed gene sets were the PGCs markers DPPA3, Thy-1, TNAP and the SSC marker Neurogenin 3 (Ngn3). The absence of Ngn3 is consistent with previous reports that Ngn3 is only expressed in adult SSC [10]. The majority of previously identified SSC genes, such as Bcl6b, Egr2, Egr3, GFRA1, and Kit Ret, showed similar expression levels in G9 and G22. Only 12 of the 50 genes were specifically up-regulated in G9 versus G22 (Figure 4A). These included Dnd1[27], GPR125 [40], Nmyc [41], ItgB1 and ItgA6, as well as Sox2, Myc, Klf4 recognized for stem cell maintenance in ESs or PGCs[42]. Surprisingly, KitL/SCF, coding for a protein secreted by Sertoli cells and involved in Spga differentiation, was up-regulated in G9 as compared to G22 [43]. This result was confirmed by immunofluorescence microscopy on Spga and Sertoli cells isolated from pre-pubertal (9 dpp) and pubertal (22 dpp) rats (Additional file 2, Figure S1). A similar observation was made by Munsie et al. [42].

Interestingly, the transcription repressor PLZF, a marker of SSCs, was expressed at higher level in G22 than in G9, suggesting that PLZF expression could reduce the proliferation rate of Spga. Indeed in hematopoietic cells, PLZF induces the G0/G1 arrest by repressing c-myc expression [44]. The other genes up-regulated in G22 (Sohlh1, Taf4b and Tex14) are known to be more expressed in differentiated type A Spga than in undifferentiated Spga [45,46].

To further evaluate the "stemness" of Spga in testis at 9 dpp, we compared our microarray data with a rat SSC gene list reported by Hamra et al. [23]. In this study 255 SSC marker genes were identified based on the microarray analysis of rat Spga with *in vitro *defined stem cell properties. We found that genes upregulated in the G9 vs G22 or the S9 vs S22 sets were significantly enriched in the SSC gene list of Hamra et al (Figure 4B, *left*); namely 61 genes were common to the set of genes up-regulated in G9 versus G22 and could be classified in ten functional categories according to their GO annotations (Figure 4B, *right*).

A large proportion of these 61 genes encode receptors, transporters, transcription factors and elements of signal transduction. Among the latter, five protein tyrosine phosphatases (PTP), namely *Dusp6*, *Ptpn14*, *Ptprg, Ptprm*, and *Ptprd*, are known to regulate adhesion, cell growth, differentiation and cell migration. The dual specific phosphatase 6 (dusp6 or MKP-3) encodes a negative feedback regulator of ERK signaling and regulates FGFR signaling during mouse development [47]. Ptpn14 and PTPrm are associated with adherent junctions and dephosphorylate key signaling substrates like beta-catenin [48] and cadherin adhesion molecules [49]. PTPrd encodes the receptor tyrosine phosphatase that regulates actin stress fibers [50]. Ptprg is transiently expressed during ES-derived embryoid body differentiation and is required for HSC lineage commitment [51]. In addition to PTPs genes, we found other genes, such as the thrombin receptor gene (*F2r*) or the fatty acid elongase (*Elovl6*), which were previously found to be differentially expressed in neuronal, embryonic and hematopoietic stem cells [52]. Thus, the G9 versus G22 gene set presented here is largely similar to the previously defined set of SSC markers. However, the SSC markers established by Hamra et al. also overlap significantly with the Sertoli-specific gene set at 9 dpp, defined by our more comprehensive approach.

### Pre-pubertal Spga and Sertoli cells specifically express genes involved in the communication between stem cells and their niche

**Figure 5:**
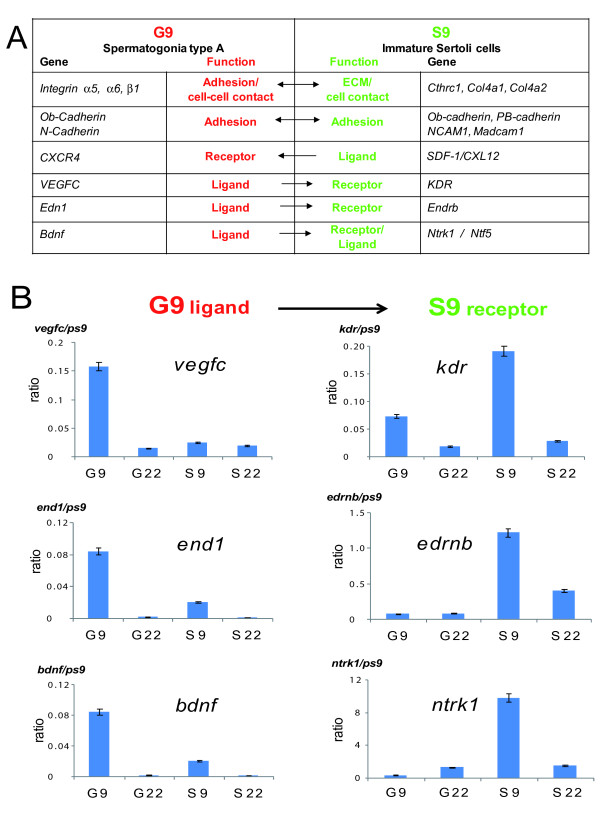
**Pre-pupertal Spga and Sertoli cells express complementary ligands and receptors known as stem cell and niche markers**. ***(A) ***Functional link associated with their gene product with a putative role in the spermatogonia stem cell niche. ***(B) ***Expression of vascular epithelial growth factor C (vegfc 1) and its receptor (kdr), of endothelin 1 (end1) and its receptor (ednrb), and of brain derived neurotrophic factor (bdnf) and its receptor (ntrk) in pre-pubertal (9 dpp) and pubertal (22 dpp) spermatogonia and Sertoli cells (G9, G22. S9, S22, respectively) was quantified by real time RT-PCR. Values were normalized to the housekeeping gene ps9; the same pattern was observed when normalizing with the expression of another housekeeping gene, ubc (not shown). Shown are the mean values (± SEM) from the 3 distinct cell preparations, for which genome-wide analysis by hybridization to a high density array is shown in Figure 2.

Stem cell niche functions are inherently linked to the communication between the niche and the stem cells. To define stem cell niche elements, we extracted the expression profiles of receptors, ligands, adhesion and extracellular proteins that are specifically up-regulated in Spga and immature Sertoli cells (Figure 5A). Some molecules are already defined as cell surface markers of SSCs, in particular integrin α6 and β1 and cadherin 1 (Cdh1), and have been associated with niche functions in pre-pubertal spermatogonia [53,54]. We found integrins α6 and β1 up-regulated in G9, but Cdh1 was not (Figure 5A). The basement membrane of the seminiferous tubule is composed of laminins, collagens, and fibronectins, ECM components that interact with integrins α6 and β1. The S9 gene set from immature Sertoli cells comprised a number of genes involved in the formation of the ECM of the basement membrane (Table 1 and Figure 5A). Interestingly, the most upregulated genes in S9 were two members of the C-type lectin gene family, *pap *and *pap3 *(pancreatitis-associated protein), which bind collagens, fibronectins and laminins. The pap/REG III family promotes cell proliferation and regeneration of pancreatic islets and may protect against apoptosis and oxidative stress [55].

The complex including the GDNF receptor GFRA1 and the receptor tyrosine kinase c-RET plays an important role in the maintenance of mouse SSC [56]. GFRA1 and GDNF were expressed both in rat Spga and in Sertoli cells, and no significant change was observed between 9 and 22 dpp (Figure 4A). This result is consistent with previous findings that GFRA1 and GDNF are expressed both in germ cells and Sertoli cells of rat testis [39]. However, although these genes are not differentially expressed in pre-pupertal versus pubertal rats, the GDNF signaling pathway is likely to be more active in pre-pubertal testis, since the downstream effector N-myc is strongly up-regulated in G9 (Table 1 and [42]).

Genes encoding the osteoblast type (Ob-Cdh, cdh11), neuronal type (N-cdh, Cdh2) or PB type (cdh22) cadherins, appeared up-regulated in G9 and S9 (Figure 5A), and Ncam1 and Madcam1 were strongly induced in S9. PB-Cdh (Cdh22) and NCAM1 (Table 1 and Figure 5A) were reported to be highly expressed in neonatal pups and down-regulated during early stages of spermatogenesis [57,58]. Both factors have been shown to interact with gonocytes in promoting SSC cell survival [57,58].

Pathways driving the development of somatic tissues (e.g. neurogenesis), may also play a role in the SSC niche (see the S9 gene sets in Figure 3). Such pathways control cell migration, proliferation or differentiation. In particular, we found the chemokine receptor CXCR4 up-regulated in G9 and its ligand SDF1/CXCL12 in S9. The SDF1/CXCL12-CXCR4 pathway is important for stem cell homing and mobilization in hematopoiesis [59]. Somatic reticular cells close to vascular endothelial cells secrete a high amount of SDF1/CXCL12 creating a vascular HSCs niche in the bone marrow different from the osteoblast niche [60].

We found other ligand-receptor couples that have a physiological role in the vascular niche specifically up-regulated in G9 and S9, such as VEGFC growth factor and receptor KDR and Endothelin 1 (End-1) and its receptor Endrb, and BDNF and NTRK or NTF5 (Figure 5A) [61]. Importantly, the receptors were upregulated in the stem cell niche S9, and their corresponding ligands were specifically up-regulated in the stem cell, G9. VEGFC and End-1 are also known to drive angiogenesis in epithelial cancers [62]. For three pairs of ligand/receptor communication we performed real time RT-PCR to quantify the relative cell type and stage-specific expression of each factor. While the ligands vegf, end1, and bdnf were significantly enriched in G9 cells, as compared to Sertoli cells or G22 cells, transcripts coding for the corresponding receptors were significantly and selectively enriched in S9 cells, as compared to all others gene sets (Figure 5B). In summary, the examples of coordinated enrichement of gene transcripts involved in the communication between SSCs and their niche corroborates the relevance of the SSC and niche-specific gene sets defined by this study.

### Common signaling pathways between SSC and their niche and testicular tumors and the tumor environment

Testicular germ cell tumors (TGCTs) originate from a precursor lesion, known as carcinoma *in situ *(CIS). CIS can be considered the neoplastic counterpart of PGCs, as they share similar morphological features, gene expression, pattern of genomic imprinting, and markers of pluripotency [28-30].

How PGCs are transformed into CIS and converted to invasive TGCT is unknown. Genomic instability of CIS might be one, but not the only contributing factor [28,63]. It has been argued that changes in the Spga microenvironment or stem cell niche could favor neoplastic transformation, and thus may lead to CIS [30,63]. Several lines of evidence support this hypothesis: first, during the formation of CIS gap junctions are disrupted, the blood-testis barrier is lost and Sertoli cells become de-differentiated [64-67]; second, the activity of the canonical SSCs signaling pathway, GDNF/c-ret is increased in CIS [68].

Our data show that the gene expression profile of pre-pubertal Sertoli cells is consistent with their presumed niche function. Consequently, de-differentiation of Sertoli cells in adult testis may reactivate this niche potential and promote SSC proliferation and neoplastic transformation towards CIS and seminoma.

To test this hypothesis, we compared the Spga (G9; G22) and the Sertoli cell (S9; S22) gene sets with the preferentially expressed gene orthologs of type II TGCTs extracted from the recent literature (Additional file 1, Table S4; [69-76]).

**Figure 6:**
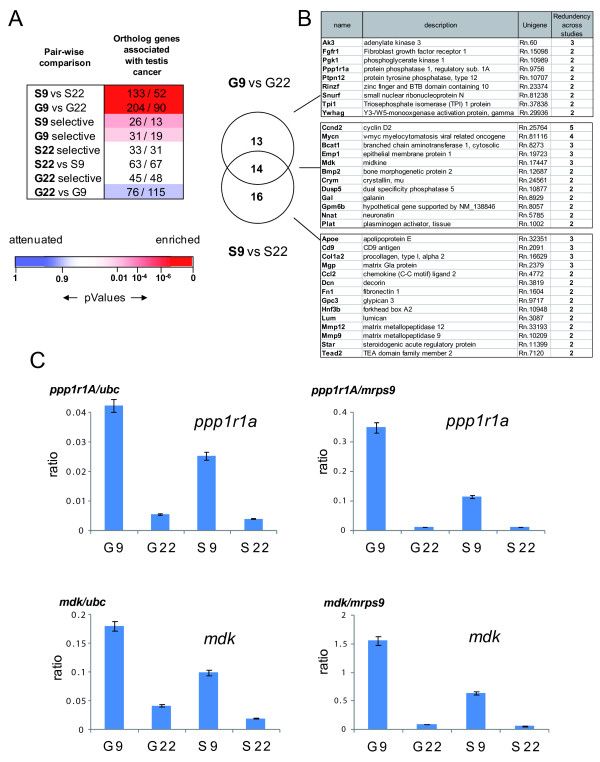
**Comparative analysis of testis cancer-associated genes with genes the pre-pubertal Spga and Sertoli cells- specific gene sets**. ***(A) ***Comparative analysis of gene transcripts enriched or attenuated in rat Spga and Sertoli cells at 9 and 22 dpp and of their human orthologs detected in several microarray studies of type II testicular germ cell tumors (TGCT). The orthologues of transcripts shown by different studies [69-76] to be enriched in TGCT were identified in the gene clusters defined in Figure 2. of Pairwise comparison between gene clusters reveals the relative enrichment or attenuation those orthologue transcripts as they are more (red) equally (white) or less (blue) abundant than expected in a random association. As an example, 133 transcripts orthologuous to genes over-expressed in TGCT[69-76] are enriched in S9 versus S22, whereas randomly 52 would be expected. The blue to red scale shows the statistical significance of enrichment or attenuation in the various clusters of genes was calculated using a Gaussian hyper-geometric test (Fischer exact probability test). ***(B) ***Genes in (A) detected in at least two independent TGCT studies, shown as a Venn diagram for the gene sets up-regulated at 9 dpp vs 22 dpp in Spga and Sertoli cells. ***(C)*** Quantitative assessment of pre-pupertal Spga and Sertoli cells gene expression for testis cancer-associated genes. Expression of protein phosphatase 1 regulatory subunit 1A (ppp1r1A), and of midkine (mdk) in pre-pubertal (9 dpp) and pubertal (22 dpp) spermatogonia and Sertoli cells (G9, G22, S9, S22, respectively) was quantified by real time RT-PCR. Values shown are normalized to two housekeeping genes ubc (right hand side) and ps9 (left hand side), respectively. Shown are the mean values (± SEM) from the 3 distinct cell preparations, for which genome-wide analysis by hybridization to a high density array is shown in Figure 2.

We compared 1436 ortholog genes, specifically upregulated in testicular cancers, with the gene sets corresponding to two-fold up-regulated transcripts of the comparisons G9 vs G22 or S9 vs S22. Significantly more testis cancer related genes, were present in those gene sets than expected by random distribution (Figure 6A). In contrast, testis cancer related genes were significantly depleted in the set of two-fold up-regulated genes in Spga at 22 dpp versus 9 dpp. These up-regulated genes were then classified, based on their redundancy in the different TGCT studies. Respectively, 27 genes of G9 vs G22 and 30 genes of S9 vs S22 were listed in two or more TGCT studies, 14 of which were up-regulated in G9 and in S9 (Figure 6B).

This latter group included CyclinD2, an early marker of CIS and important for transformation of germ cell tumors [77,78], and Mycn, a proto-oncogene effector downstream of GNDF/GRFa1 favoring proliferation of SSCs [41]. Interestingly, the overlap of the TGCT-specific genes with S9 comprised a large number of genes encoding secreted proteins (Ccl2, MMP9, MMP12, Plat) or ECM proteins (collagen type I, decorin, fibronectin 1, lumican), which are considered important for the somatic mammalian stem cell niches [79]. Other factors, like the matrix Gla protein or Glypican 3, are known to repress differentiation pathways such as BMP2/4 [80] and Hedgehog [81].

We confirmed the specific and significant enrichment of gene transcripts common to G9 and testicular cancer or to S9 and tumor environment by real time RT-PCR. Both candidates tested, protein phosphatase 1 regulatory 1A (Ppp1r1a) and midkine (mdk), were more expressed in G9 cells than in S9 cells and showed very little expression in G22 and S22 cells (Figure 6B).

These results suggest that the gene expression programs of SSC and their niches, may be reactivated and amplified in testicular cancer. Further studies in TGCTs might validate the candidate genes emerging from this study as potential prognostic markers and/or targets for treatment of early TGCT.

## Discussion

Spermatogenesis is a highly organized process initiated at puberty in mammalian species. Before puberty, a massive increase of mitotic Spga supports the onset of spermatogenesis. Newly formed Sertoli cells sustain this proliferation of Spga, but also maintain the potential of extending the stem cell niche for dividing SSCs. This work was based on the hypothesis that in rodents, a larger fraction of Spga with stem cell potential exists at pre-puberty than at puberty. Furthermore, we hypothesized that pre-pubertal Sertoli cells might fulfill SSC niche functions. Indeed, previous studies supported these hypotheses. First, transplantation studies of donor germ cells in recipient animals show that engraftment of Spga from pre-pubertal rodents have higher success rates than Spga from adult animals [16-18]. Similarly, engrafted donor germ cells develop better in recipient pups than adults [19,20]. Second, the fraction of mitotic Spga with the potential of self-renewal is bigger than adult SSCs population [10,11,13].

Indeed, as Yoshida and colleagues have elegantly shown, transiently amplifying Spga are able to function as stem cells upon transplantation, provided that they enter an appropriate SSC niche [11,13]. Thus mammals, in contrast to e.g. *Drosophila*, can compensate for the loss of germ stem cells that may occur during the reproductive life. The downside of this plasticity is that the inappropriate favoring of stem-ness, due to the expression of relevant factors by Spga or Sertoli cells, may lead to testis cancer. To understand the mechanisms of SSC maintenance, it is essential to investigate the interactions between Spga and Sertoli cells. Here we report a first approach towards this goal, based on differential gene expression profiles for pre-pubertal and pubertal Spga and Sertoli cells, and defined transcript sets that characterize stem-ness and niche properties.

Our approach was corroborated by the fact that the pre-pubertal gene sets included, as expected, known general stem cell markers, SSC markers, as well as known niche markers (Figure 4). Importantly, genes coding for products that mediate cell to cell communication were predominantly upregulated in SSCs and the niche. The pre-pubertal gene sets also showed a highly significant overlap with genes over-expressed in testis cancer. And again, these overlapping gene lists highlighted the importance of stem cell to niche communication.

Mammals maintain differentiated stem cell reservoirs throughout life. Location, cell number and replication of these reservoirs are strictly controlled. Stem cells are confined to their respective niches by a system of "checks and balances" established between stem cells and niche cells. This system appears to be based upon reciprocal activation of intracellular signaling cascades involving secreted factors and their receptors, ion channels, transporters, protein kinases and phosphatases, transcription factors, and cell cycle control elements. Given the large number of genes coding for such elements, each specific stem cell niche ensemble is controlled by common as well as distinct elements. In this study we defined gene sets for SSCs and their niche. The most likely candidates of the extra-cellular elements contributing to such control are those listed in Figure 5A.

It further appears that the system is refined to assure proper localization of stem cell niches. Very elegant work by Yoshida and co-workers demonstrated the SSC niche within the vasculature [82], which is most likely based upon the inclusion of endothelial derived growth factors in the system controlling SSCs.

To further validate the relevance of specific genes as coding for stem cell niche factors or differentiation factors in spermatogenesis, RNAi-based gene silencing in Spga and Sertoli cells co-cultures may be a first approach. Transplantation of genetically modified donor-derived germ cells in recipient testes could later definitively confirm the stem cell activity of the specific candidate genes.

Uncontrolled SSCs have the potential to develop into testis cancer. Testicular germ cell tumours (TGCTs) typically occur in adolescents and adults. These tumors are the most frequently diagnosed cancer in Caucasian adolescents and young adults. The precursor of type II GCTs (the most frequent one) originates from a PGC/gonocyte, i.e. an embryonic cell [28-30]. The highly significant overlap between gene expression of pre-pubertal Spga and Sertoli cells with gene expression in testis cancer (Figure 6) supports evidence that stem cell niche interactions are maintained in testis cancer. Indeed, the large majority of the genes over-expressed in pre-pubertal Spga and Sertoli cells, orthologs of which have also elevated expression level in testis cancer, code for the proteins involved in cell to cell communication.

Cell to cell communication is obviously an important feature and is deregulated in most cancers. However, we find factors that are likely to be important for stem cell to niche communication which are also upregulated in cancers that are likely to have derived from deregulated stem cells.

The replication of expression patterns that characterize stem cell-niche interactions in the tumor and surrounding stroma suggests that the concept of mutual gene programming through extensive communication is important for the understanding of tumorigenesis. This concept is in line with and provides an explanation for field effects, i.e. the fact that testis and other cancers progresse in a specific "permissive" tissue environment [83].

## Conclusions

The present study provides a large list of candidate genes, the expression of which characterizes the specific cellular interactions occurring between Spga and Sertoli cells, which reciprocally control their proliferation and differentiation during puberty. Specifically, SSCs and pre-pubertal Sertoli cells which establish the SSC niche were shown to express complementary factors and receptors for their communication. These same factors and the corresponding signaling pathways most likely have their significance in the development of testicular cancer, and notably in the communication between tumor cells and their micro-environment.

## Methods

### Cell and RNA purification

To isolate Spga and Sertoli cells, testes of 45 rats at 9 dpp and 20 rats at 22 dpp were excised and decapsulated. The procedure of cell purification is depicted in Additional file 2, Figure S2 and was adapted from previous publications [84-86]. Seminiferous tubules were first isolated from surrounding interstitial cells by collagenase dispase treatement. After three sedimentation steps, the tubules were separated in two fractions. To purify Spga, a fraction of seminiferous tubules was treated with trypsin, then subjected to centrifugal elutriation to collect the diploid cells. The diploid cells were then resuspended in a minimal medium (15 mM Hepes buffered F12/DMEM supplemented with 20 μg/ml gentallin, 20 U/ml nystatin, 1.2 g/L sodium bicarbonate, 10 μg/ml insulin, 10 μg/ml human transferrin, 0.2% serum) and subjected to differential plating for 15 hours. Floating Spga cells were collected by centrifugation and directly frozen in liquid nitrogen. To purify the somatic supporting cells, half the fraction of tubules was treated with collagenase dispase, then, differential plating was directly performed to collect the corresponding adherent Sertoli cells. In the germ cell fractions, 78 ± 2% of the cells were vimentin negative and their viability was 93 ± 1%. In the Sertoli cell fraction, 76.5 ± 3.0% of the cells were stained positive with an antibody against vimentin [87-89]; their viability was 82 ± 5%.

Total RNA was isolated from frozen cells using Trizol (Invitrogen), then, a subsequent purification step was performed using the RNAeasy kit (Qiagen; Hombrechtikon, CH). The purity of the total RNA was analyzed using an Agilent Bioanalyzer RNA Chip (Agilent Inc. Paolo Alto, CA).

### Immunohistochemistry

Immuno-histochemistry was performed following established procedures [90,91] on sections of rat testis from 9 dpp and 22 dpp using antibodies to PCNA (DAKO).

### Microarray probe labelling and hybridization

A small-scale protocol from Affymetrix (High Wycombe, UK) was used to reproducibly amplify and label total RNA. 100 ng total RNA were converted into double-stranded cDNA using a cDNA synthesis kit (Superscript; Invitrogen Corp., Carlsbad, CA) with a special oligo(dT)_24 primer containing a T7 RNA promoter site. After the first cRNA amplification by *in vitro *transcription using the Ambion MEGAscript T7 kit (Ambion, Austin, TX), 400 ng cRNA were once more reverse transcribed, and biotinylated cRNAs were generated from double-strand cDNAs using an *in vitro *transcription labeling kit from Affymetrix. For each probe, 20 μg of the second amplification biotinylated cRNA were fragmented and hybridized to Affimetrix rat expression array 230 2.0 (Affymetrix; Santa Clara, CA) following standard protocols. Three independent sets of total RNA were extracted from purified cells prepared on different days on distinct pools of animals. For each condition, the three independent sets of total RNA were purified and used as template for probe generation. These triplicates preparations were performed to define biological variability between the samples. GeneChips were incubated at 45°C for 16 h with biotin-labeled cRNAs probes, and then washed and stained using a streptavidin- phycoerythrin conjugate with antibody amplification as described in the protocol from Affymetrix, using Affymetrix GeneChip Fluidics Station 450. GeneChips were scanned on a GCS3000 scanner (Affymetrix, Santa Clara, CA).

### Selection of differentially expressed genes

To identify differentially expressed transcripts, pairwise comparison analyses were carried out with Affymetrix GCOS 1.2. Each of the experimental samples (n = 3) was compared with each of the reference samples (n = 3), resulting in nine pairwise comparisons. This approach, which is based on the Mann-Whitney pairwise comparison test, allows the ranking of results by concordance, as well as the calculation of significance (P value) of each identified change in gene expression [92]. Genes for which the concordance in the pairwise comparisons exceeded a threshold (e.g., 60%) were considered to be statistically significant. A 77% cutoff in consistency of change (at least 7 of 9 comparisons were either increased or decreased) was then applied to identify potential dimorphic-regulated genes. Only genes that satisfied the pairwise comparison test and displayed two-fold change in expression were selected for further study. This conservative analytical approach was used to limit the number of false-positives. Regulated genes were organized and visualized using the GeneSpring software (Agilent Inc., Paolo Alto, CA).

### Cluster and Gene Ontology (GO) analysis

Category enrichment or depletion was performed by collecting the observed number of transcripts and respective hypergeometric P-values for each category using Genespring. The expected number of transcripts was calculated based on the total number of annotated RAE230 transcripts in the Gene ontology consortium (5727), the number of annotated transcripts in the corresponding gene ontology category, and the fraction of transcripts in the cluster present in the category. GO category enrichment or depletion were displayed as in Chalmel et al [21] for each respective cluster. A GO category was considered as enriched in a group of transcripts if the P value was <0.001 and the number of observed transcripts in the cluster was >3. P values close to 0 indicate significant enrichment whereas P values close to 1 represent significant depletion.

All gene expression array data are avaible at Gene Expression Omnibus (GEO; http://www.ncbi.nlm.nih.gov/projects/geo/).

#### Real time RT-PCR

Quantification of gene expression was obtained by real-time RT-PCR performed essentially as described earlier [93,94] using SYBR green and the following forward (F) and reverse (R) primer pairs: **plk2 (NM031821) **F: GGGCAAGGGTGGATTTG R: GCGTAGACTTTGTTGTTTGTCAGATC, for **bdnf (NM012513) **F: ACTGTCCTGCTACCGCAGTTG R: GGGTCGCAGAACCGCTAAA, **gap43 (NM017195) **F: TGCAGAAAGCAGCCAAGCT R: CGGGCACTTTCCTTAGGTTTG, **ntrk1 (NM021589) **F:ACGGAGCTCTATGTGGAAAACC R: CCCTGCAGGTCCTCAAACTC, **pap3 (L20869) **F:GGCTCCTATTGCTATGCCTTGT R: CAGGCCAGATCTGCATCAAA, **ppp1r1a (NM022676) **F:CCAGCACAGAGGACCTTTCAG R:TCAGACCAAGCTGGCTCCTT, **vegfc (NM053653) **F:GCGAGGTCAAGGGTTTCGA R: TGAGCTCATCTACACTGGACACAGA, **mdk (NM030859) **F:GCGCATCCATTGCAAGGT R: TGCAGTCGGCTCCAAACTC, **kdr (U93307) **F: TTCCGTCCGGACTCTTACGT R: GCAAGCTGCGTCATTTCCTT, and **house-keeping genes endothelin 1 (NM012548) **F:GATTATTTTCCCGTGATCTTCTCTCT R: TGCTCCCAAGACAGCTGTTTC, **ubc (NM017314) **F:TCGTACCTTTCTCACCACAGTATCTAG R: GAAAACTAAGACACCTCCCCATCA, **edrnb (X57764) **F:GGTATGCAGATTGCCTTGAATG R: GCAGAATACTGTCTTGGCCACTT, and **mrps9 (NM001100549) **F:TGATGTTCCCTTTCCACTTCCT R: TCCCTCCCCCGGAGACT.

## Authors' contributions

SR carried out the RNA purification and array study, data assembly and analysis, drafted, wrote and corrected the manuscript, and approved its final version. DG performed data mining and interpretation of expression data from testicular tumors and tumor icroenvironment and the comparison with gene expression of germ cells and Sertoli cells, and approved the final version of manuscript. MV was essential for preparing the specific cell preparations of different cell types and stages and approved the final version of manuscript. PT made substantial contributions to the conception, design, data analysis, and funding of the project, and approved the final version of manuscript. YQZ performed immunohistochemistry for expression studies of relevant genes and approved the final version of the manuscript. WS designed the real-time PCR, in drafted and revised the manuscript, and approved final version of the manuscript. PD was involved in the development of novel critical purification methods for spematogonia stem cells, in study conception and design, provision of study material, and approved the final version of the manuscript. IIF designed the concept and supervised the study, was essential in funding of the project, and drafted, wrote and corrected the manuscript, and approved final version of manuscript.

## Supplementary Material

Additional file 1 **Methods and validation of cell purification**. Figures S1 and S2 and explanations of Tables S1-S4. Click here for file

Additional file 2 **Updated gene lists Description: Tables S1-S4**. Click here for file
