# Supplementary material for: Gene expression profiling of rat spermatogonia and Sertoli cells reveals signaling pathways from stem cells to niche and testicular cancer cells to surrounding stroma
Source: BMC Genomics. 2011 Jan 13;12:29. doi: 10.1186/1471-2164-12-29 (PMC3033334; doi:10.1186/1471-2164-12-29)
Supplement: Additional file 1 — Methods and validation of cell purification. Figures S1 and S2 and explanations of Tables S1-S4. [file 1471-2164-12-29-S1.PPTX]

## Slide 1
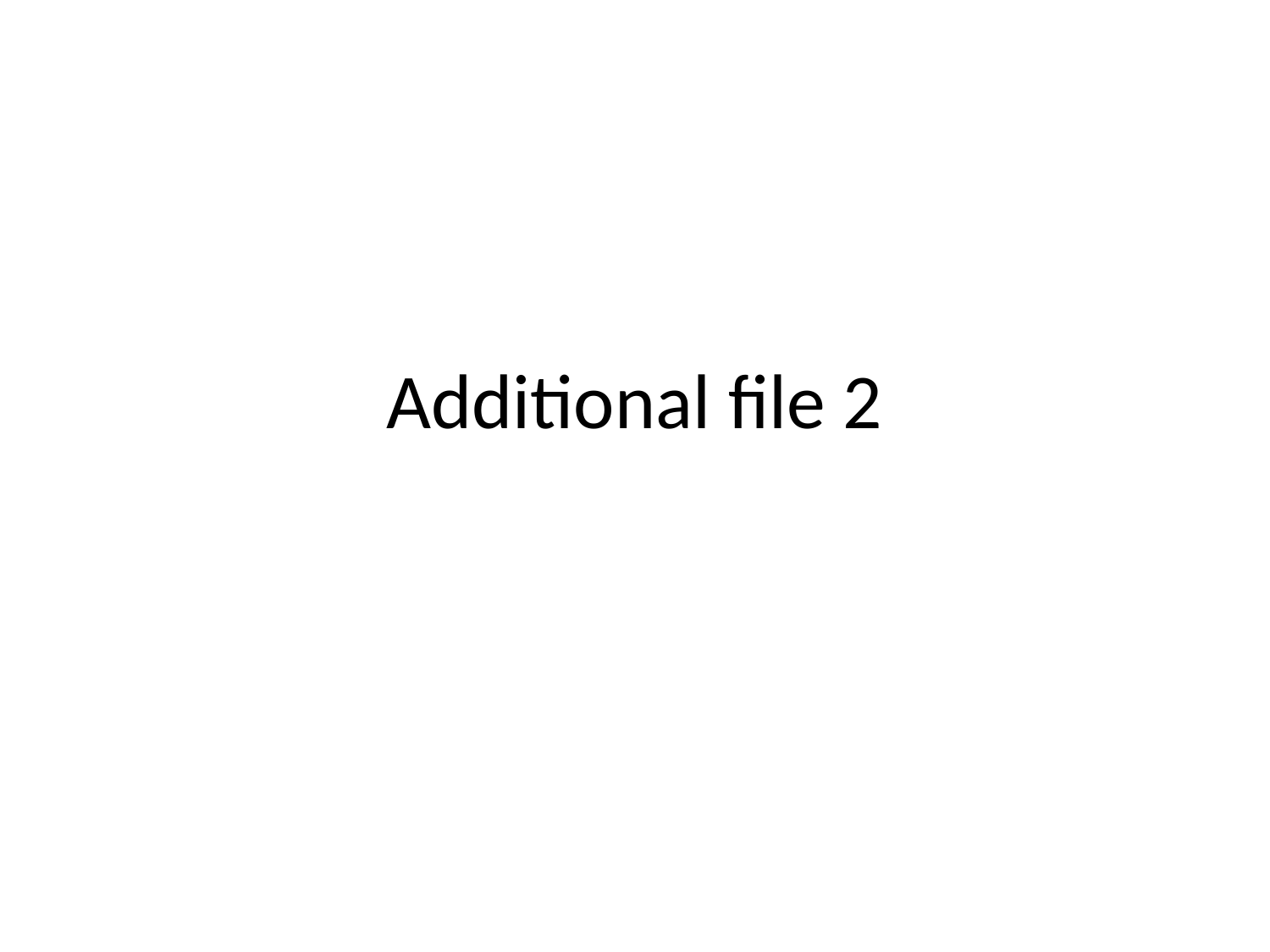

# Additional file 2

## Slide 2
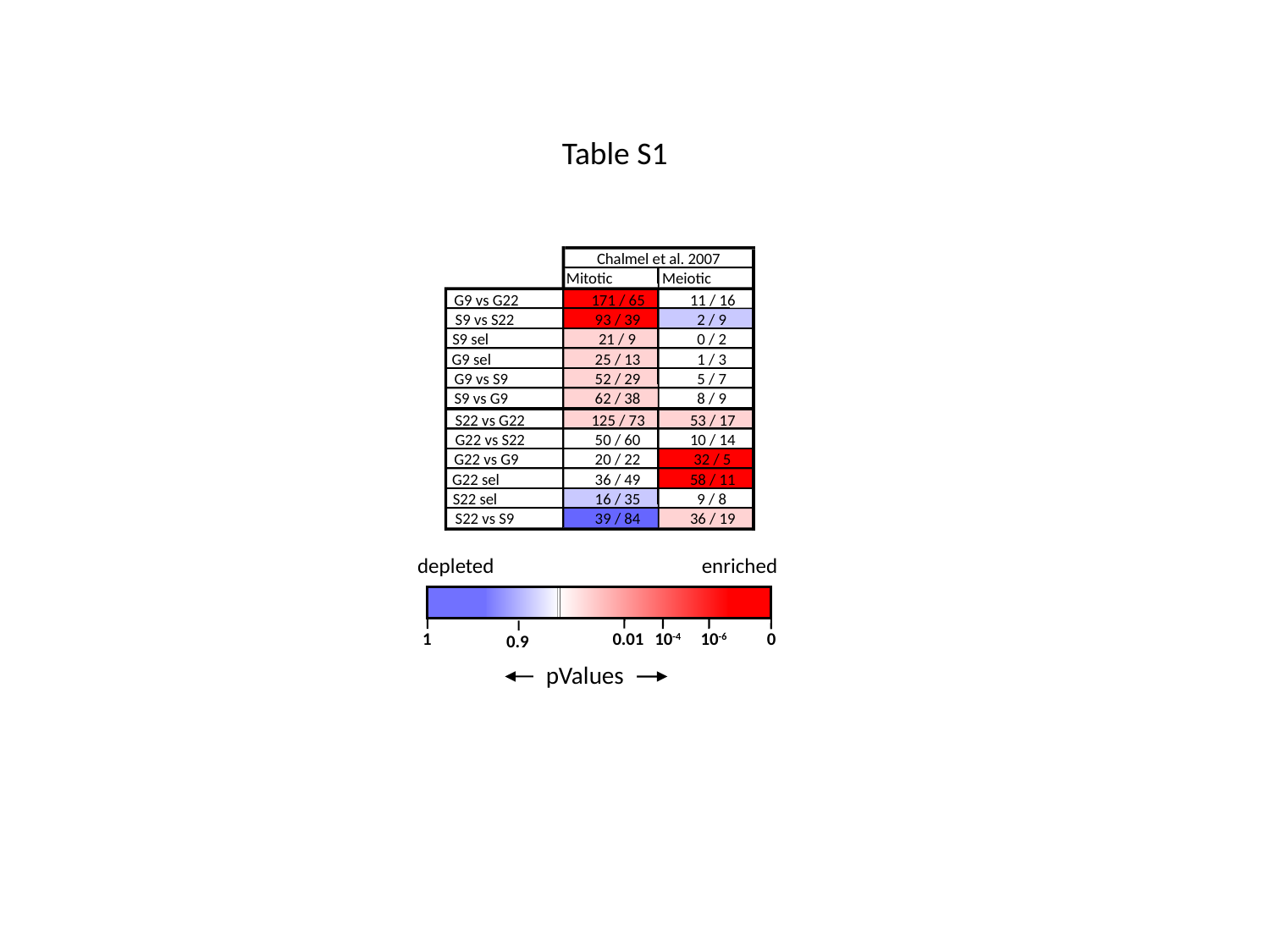

Table S1
Chalmel et al. 2007
Mitotic
Meiotic
G9 vs G22
171 / 65
11 / 16
S9 vs S22
93 / 39
2 / 9
S9 sel
21 / 9
0 / 2
G9 sel
25 / 13
1 / 3
G9 vs S9
52 / 29
5 / 7
S9 vs G9
62 / 38
8 / 9
S22 vs G22
125 / 73
53 / 17
G22 vs S22
50 / 60
10 / 14
G22 vs G9
20 / 22
32 / 5
G22 sel
36 / 49
58 / 11
S22 sel
16 / 35
9 / 8
S22 vs S9
39 / 84
36 / 19
depleted
enriched
1
0.01
10-4
10-6
0
0.9
pValues

## Slide 3
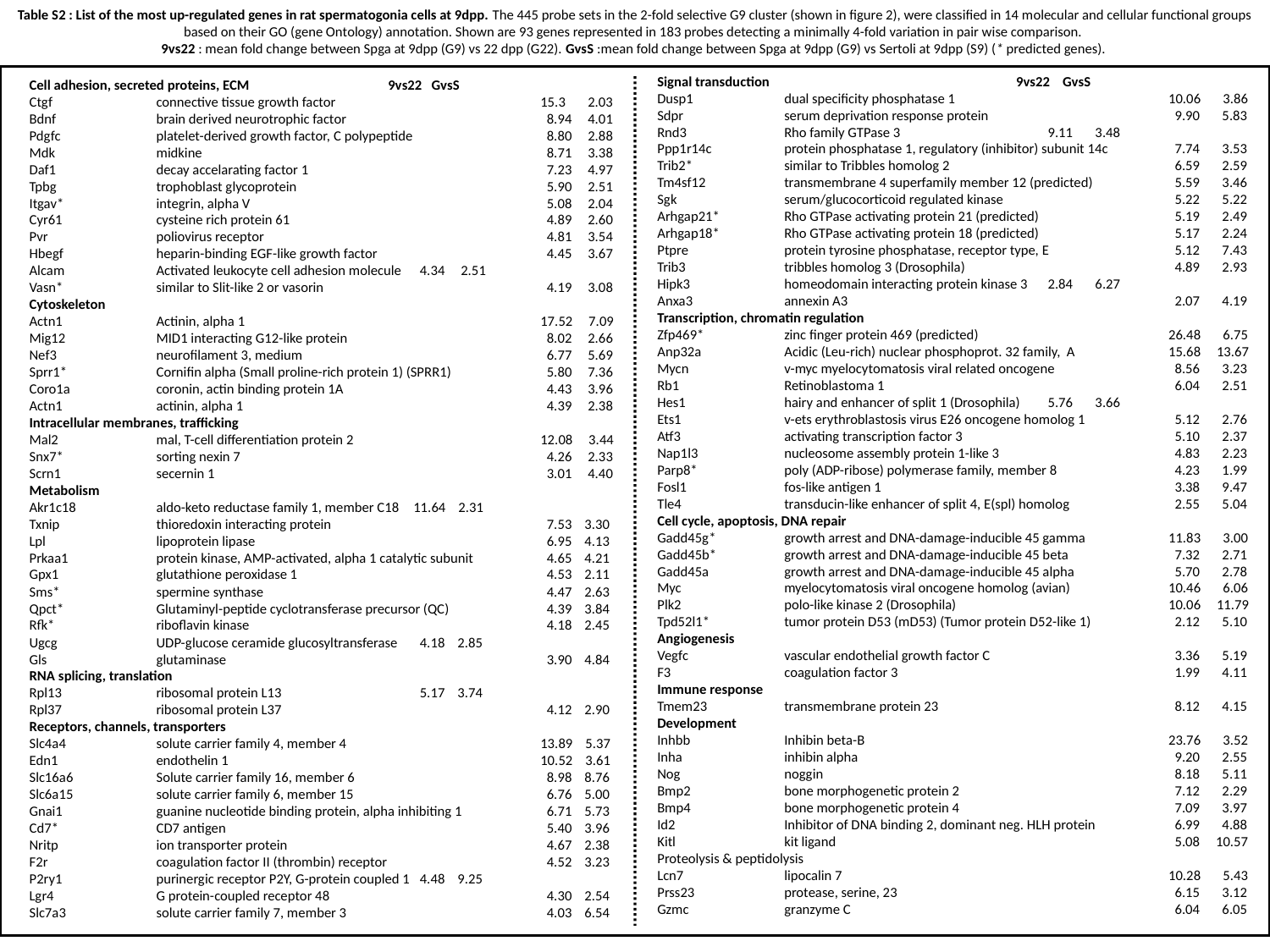

Table S2 : List of the most up-regulated genes in rat spermatogonia cells at 9dpp. The 445 probe sets in the 2-fold selective G9 cluster (shown in figure 2), were classified in 14 molecular and cellular functional groups based on their GO (gene Ontology) annotation. Shown are 93 genes represented in 183 probes detecting a minimally 4-fold variation in pair wise comparison.
9vs22 : mean fold change between Spga at 9dpp (G9) vs 22 dpp (G22). GvsS :mean fold change between Spga at 9dpp (G9) vs Sertoli at 9dpp (S9) (* predicted genes).
Signal transduction		 9vs22 GvsS
Dusp1	dual specificity phosphatase 1		 10.06 3.86
Sdpr	serum deprivation response protein		 9.90 5.83
Rnd3	Rho family GTPase 3		 9.11 3.48
Ppp1r14c	protein phosphatase 1, regulatory (inhibitor) subunit 14c	 7.74 3.53
Trib2*	similar to Tribbles homolog 2		 6.59 2.59
Tm4sf12	transmembrane 4 superfamily member 12 (predicted)	 5.59 3.46
Sgk	serum/glucocorticoid regulated kinase		 5.22 5.22
Arhgap21*	Rho GTPase activating protein 21 (predicted)	 5.19 2.49
Arhgap18*	Rho GTPase activating protein 18 (predicted)	 5.17 2.24
Ptpre	protein tyrosine phosphatase, receptor type, E	 5.12 7.43
Trib3	tribbles homolog 3 (Drosophila)		 4.89 2.93
Hipk3	homeodomain interacting protein kinase 3	 2.84 6.27
Anxa3	annexin A3			 2.07 4.19
Transcription, chromatin regulation
Zfp469*	zinc finger protein 469 (predicted)		 26.48 6.75
Anp32a	Acidic (Leu-rich) nuclear phosphoprot. 32 family, A	 15.68 13.67
Mycn	v-myc myelocytomatosis viral related oncogene	 8.56 3.23
Rb1	Retinoblastoma 1			 6.04 2.51
Hes1	hairy and enhancer of split 1 (Drosophila)	 5.76 3.66
Ets1	v-ets erythroblastosis virus E26 oncogene homolog 1	 5.12 2.76
Atf3	activating transcription factor 3		 5.10 2.37
Nap1l3	nucleosome assembly protein 1-like 3		 4.83 2.23
Parp8*	poly (ADP-ribose) polymerase family, member 8	 4.23 1.99
Fosl1	fos-like antigen 1			 3.38 9.47
Tle4	transducin-like enhancer of split 4, E(spl) homolog	 2.55 5.04
Cell cycle, apoptosis, DNA repair
Gadd45g*	growth arrest and DNA-damage-inducible 45 gamma	 11.83 3.00
Gadd45b*	growth arrest and DNA-damage-inducible 45 beta	 7.32 2.71
Gadd45a	growth arrest and DNA-damage-inducible 45 alpha	 5.70 2.78
Myc	myelocytomatosis viral oncogene homolog (avian)	 10.46 6.06
Plk2	polo-like kinase 2 (Drosophila)		 10.06 11.79
Tpd52l1*	tumor protein D53 (mD53) (Tumor protein D52-like 1)	 2.12 5.10
Angiogenesis
Vegfc	vascular endothelial growth factor C		 3.36 5.19
F3	coagulation factor 3			 1.99 4.11
Immune response
Tmem23	transmembrane protein 23		 8.12 4.15
Development
Inhbb	Inhibin beta-B			 23.76 3.52
Inha	inhibin alpha			 9.20 2.55
Nog	noggin			 8.18 5.11
Bmp2	bone morphogenetic protein 2		 7.12 2.29
Bmp4	bone morphogenetic protein 4		 7.09 3.97
Id2	Inhibitor of DNA binding 2, dominant neg. HLH protein	 6.99 4.88
Kitl	kit ligand			 5.08 10.57
Proteolysis & peptidolysis
Lcn7	lipocalin 7			 10.28 5.43
Prss23	protease, serine, 23			 6.15 3.12
Gzmc	granzyme C			 6.04 6.05
Cell adhesion, secreted proteins, ECM 	 9vs22 GvsS
Ctgf	connective tissue growth factor		 15.3 2.03
Bdnf	brain derived neurotrophic factor		 8.94 4.01
Pdgfc	platelet-derived growth factor, C polypeptide 	 8.80 2.88
Mdk	midkine			 8.71 3.38
Daf1	decay accelarating factor 1		 7.23 4.97
Tpbg	trophoblast glycoprotein		 5.90 2.51
Itgav*	integrin, alpha V			 5.08 2.04
Cyr61	cysteine rich protein 61		 4.89 2.60
Pvr	poliovirus receptor			 4.81 3.54
Hbegf	heparin-binding EGF-like growth factor		 4.45 3.67
Alcam	Activated leukocyte cell adhesion molecule	 4.34 2.51
Vasn*	similar to Slit-like 2 or vasorin		 4.19 3.08
Cytoskeleton
Actn1	Actinin, alpha 1			 17.52 7.09
Mig12	MID1 interacting G12-like protein		 8.02 2.66
Nef3	neurofilament 3, medium		 6.77 5.69
Sprr1*	Cornifin alpha (Small proline-rich protein 1) (SPRR1)	 5.80 7.36
Coro1a	coronin, actin binding protein 1A		 4.43 3.96
Actn1	actinin, alpha 1			 4.39 2.38
Intracellular membranes, trafficking
Mal2	mal, T-cell differentiation protein 2		 12.08 3.44
Snx7*	sorting nexin 7			 4.26 2.33
Scrn1	secernin 1			 3.01 4.40
Metabolism
Akr1c18	aldo-keto reductase family 1, member C18	 11.64 2.31
Txnip	thioredoxin interacting protein		 7.53 3.30
Lpl	lipoprotein lipase			 6.95 4.13
Prkaa1	protein kinase, AMP-activated, alpha 1 catalytic subunit	 4.65 4.21
Gpx1	glutathione peroxidase 1		 4.53 2.11
Sms*	spermine synthase			 4.47 2.63
Qpct*	Glutaminyl-peptide cyclotransferase precursor (QC)	 4.39 3.84
Rfk*	riboflavin kinase			 4.18 2.45
Ugcg	UDP-glucose ceramide glucosyltransferase	 4.18 2.85
Gls	glutaminase			 3.90 4.84
RNA splicing, translation
Rpl13	ribosomal protein L13		 5.17 3.74
Rpl37	ribosomal protein L37 		 4.12 2.90
Receptors, channels, transporters
Slc4a4	solute carrier family 4, member 4		 13.89 5.37
Edn1	endothelin 1			 10.52 3.61
Slc16a6	Solute carrier family 16, member 6		 8.98 8.76
Slc6a15	solute carrier family 6, member 15		 6.76 5.00
Gnai1	guanine nucleotide binding protein, alpha inhibiting 1	 6.71 5.73
Cd7*	CD7 antigen			 5.40 3.96
Nritp	ion transporter protein		 4.67 2.38
F2r	coagulation factor II (thrombin) receptor		 4.52 3.23
P2ry1	purinergic receptor P2Y, G-protein coupled 1	 4.48 9.25
Lgr4 	G protein-coupled receptor 48		 4.30 2.54
Slc7a3	solute carrier family 7, member 3		 4.03 6.54

## Slide 4
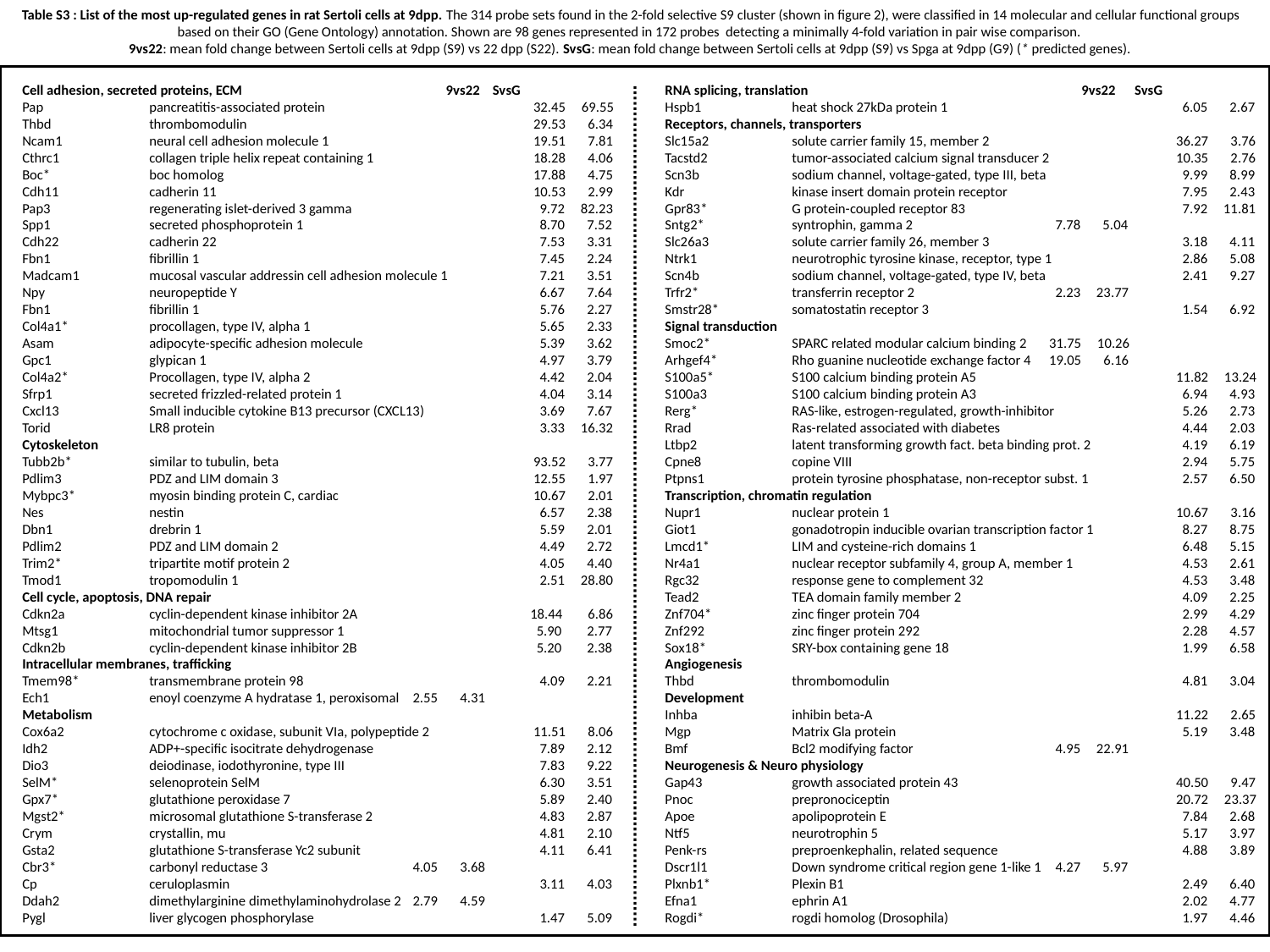

Table S3 : List of the most up-regulated genes in rat Sertoli cells at 9dpp. The 314 probe sets found in the 2-fold selective S9 cluster (shown in figure 2), were classified in 14 molecular and cellular functional groups based on their GO (Gene Ontology) annotation. Shown are 98 genes represented in 172 probes detecting a minimally 4-fold variation in pair wise comparison.
9vs22: mean fold change between Sertoli cells at 9dpp (S9) vs 22 dpp (S22). SvsG: mean fold change between Sertoli cells at 9dpp (S9) vs Spga at 9dpp (G9) (* predicted genes).
Cell adhesion, secreted proteins, ECM 9vs22 SvsG
Pap	pancreatitis-associated protein		 32.45 69.55
Thbd	thrombomodulin			 29.53 6.34
Ncam1	neural cell adhesion molecule 1		 19.51 7.81
Cthrc1	collagen triple helix repeat containing 1		 18.28 4.06
Boc*	boc homolog			 17.88 4.75
Cdh11	cadherin 11			 10.53 2.99
Pap3	regenerating islet-derived 3 gamma		 9.72 82.23
Spp1	secreted phosphoprotein 1		 8.70 7.52
Cdh22	cadherin 22			 7.53 3.31
Fbn1	fibrillin 1			 7.45 2.24
Madcam1	mucosal vascular addressin cell adhesion molecule 1	 7.21 3.51
Npy	neuropeptide Y			 6.67 7.64
Fbn1	fibrillin 1			 5.76 2.27
Col4a1*	procollagen, type IV, alpha 1		 5.65 2.33
Asam	adipocyte-specific adhesion molecule		 5.39 3.62
Gpc1	glypican 1			 4.97 3.79
Col4a2*	Procollagen, type IV, alpha 2 		 4.42 2.04
Sfrp1	secreted frizzled-related protein 1		 4.04 3.14
Cxcl13	Small inducible cytokine B13 precursor (CXCL13) 	 3.69 7.67
Torid	LR8 protein			 3.33 16.32
Cytoskeleton
Tubb2b*	similar to tubulin, beta 		 93.52 3.77
Pdlim3	PDZ and LIM domain 3		 12.55 1.97
Mybpc3*	myosin binding protein C, cardiac 		 10.67 2.01
Nes	nestin			 6.57 2.38
Dbn1	drebrin 1			 5.59 2.01
Pdlim2	PDZ and LIM domain 2		 4.49 2.72
Trim2*	tripartite motif protein 2		 4.05 4.40
Tmod1	tropomodulin 1			 2.51 28.80
Cell cycle, apoptosis, DNA repair
Cdkn2a	cyclin-dependent kinase inhibitor 2A		18.44 6.86
Mtsg1	mitochondrial tumor suppressor 1		 5.90 2.77
Cdkn2b	cyclin-dependent kinase inhibitor 2B 		 5.20 2.38
Intracellular membranes, trafficking
Tmem98*	transmembrane protein 98		 4.09 2.21
Ech1	enoyl coenzyme A hydratase 1, peroxisomal	 2.55 4.31
Metabolism
Cox6a2	cytochrome c oxidase, subunit VIa, polypeptide 2	 11.51 8.06
Idh2	ADP+-specific isocitrate dehydrogenase		 7.89 2.12
Dio3	deiodinase, iodothyronine, type III		 7.83 9.22
SelM*	selenoprotein SelM			 6.30 3.51
Gpx7*	glutathione peroxidase 7 		 5.89 2.40
Mgst2*	microsomal glutathione S-transferase 2 		 4.83 2.87
Crym	crystallin, mu			 4.81 2.10
Gsta2	glutathione S-transferase Yc2 subunit		 4.11 6.41
Cbr3*	carbonyl reductase 3 		 4.05 3.68
Cp	ceruloplasmin			 3.11 4.03
Ddah2	dimethylarginine dimethylaminohydrolase 2	 2.79 4.59
Pygl	liver glycogen phosphorylase		 1.47 5.09
RNA splicing, translation 9vs22 SvsG
Hspb1	heat shock 27kDa protein 1		 6.05 2.67
Receptors, channels, transporters
Slc15a2	solute carrier family 15, member 2		 36.27 3.76
Tacstd2	tumor-associated calcium signal transducer 2	 10.35 2.76
Scn3b	sodium channel, voltage-gated, type III, beta	 9.99 8.99
Kdr	kinase insert domain protein receptor		 7.95 2.43
Gpr83*	G protein-coupled receptor 83 		 7.92 11.81
Sntg2*	syntrophin, gamma 2 		 7.78 5.04
Slc26a3	solute carrier family 26, member 3		 3.18 4.11
Ntrk1	neurotrophic tyrosine kinase, receptor, type 1	 2.86 5.08
Scn4b	sodium channel, voltage-gated, type IV, beta	 2.41 9.27
Trfr2*	transferrin receptor 2 		 2.23 23.77
Smstr28*	somatostatin receptor 3 		 1.54 6.92
Signal transduction
Smoc2*	SPARC related modular calcium binding 2 	 31.75 10.26
Arhgef4* 	Rho guanine nucleotide exchange factor 4 	 19.05 6.16
S100a5*	S100 calcium binding protein A5 		 11.82 13.24
S100a3	S100 calcium binding protein A3		 6.94 4.93
Rerg*	RAS-like, estrogen-regulated, growth-inhibitor	 5.26 2.73
Rrad	Ras-related associated with diabetes		 4.44 2.03
Ltbp2	latent transforming growth fact. beta binding prot. 2	 4.19 6.19
Cpne8	copine VIII			 2.94 5.75
Ptpns1	protein tyrosine phosphatase, non-receptor subst. 1	 2.57 6.50
Transcription, chromatin regulation
Nupr1	nuclear protein 1			 10.67 3.16
Giot1	gonadotropin inducible ovarian transcription factor 1	 8.27 8.75
Lmcd1*	LIM and cysteine-rich domains 1 		 6.48 5.15
Nr4a1	nuclear receptor subfamily 4, group A, member 1	 4.53 2.61
Rgc32	response gene to complement 32		 4.53 3.48
Tead2	TEA domain family member 2		 4.09 2.25
Znf704*	zinc finger protein 704		 2.99 4.29
Znf292	zinc finger protein 292		 2.28 4.57
Sox18*	SRY-box containing gene 18 		 1.99 6.58
Angiogenesis
Thbd	thrombomodulin			 4.81 3.04
Development
Inhba	inhibin beta-A			 11.22 2.65
Mgp	Matrix Gla protein			 5.19 3.48
Bmf	Bcl2 modifying factor		 4.95 22.91
Neurogenesis & Neuro physiology
Gap43	growth associated protein 43		 40.50 9.47
Pnoc	prepronociceptin			 20.72 23.37
Apoe	apolipoprotein E			 7.84 2.68
Ntf5	neurotrophin 5			 5.17 3.97
Penk-rs	preproenkephalin, related sequence		 4.88 3.89
Dscr1l1	Down syndrome critical region gene 1-like 1	 4.27 5.97
Plxnb1*	Plexin B1 			 2.49 6.40
Efna1	ephrin A1			 2.02 4.77
Rogdi*	rogdi homolog (Drosophila)		 1.97 4.46

## Slide 5
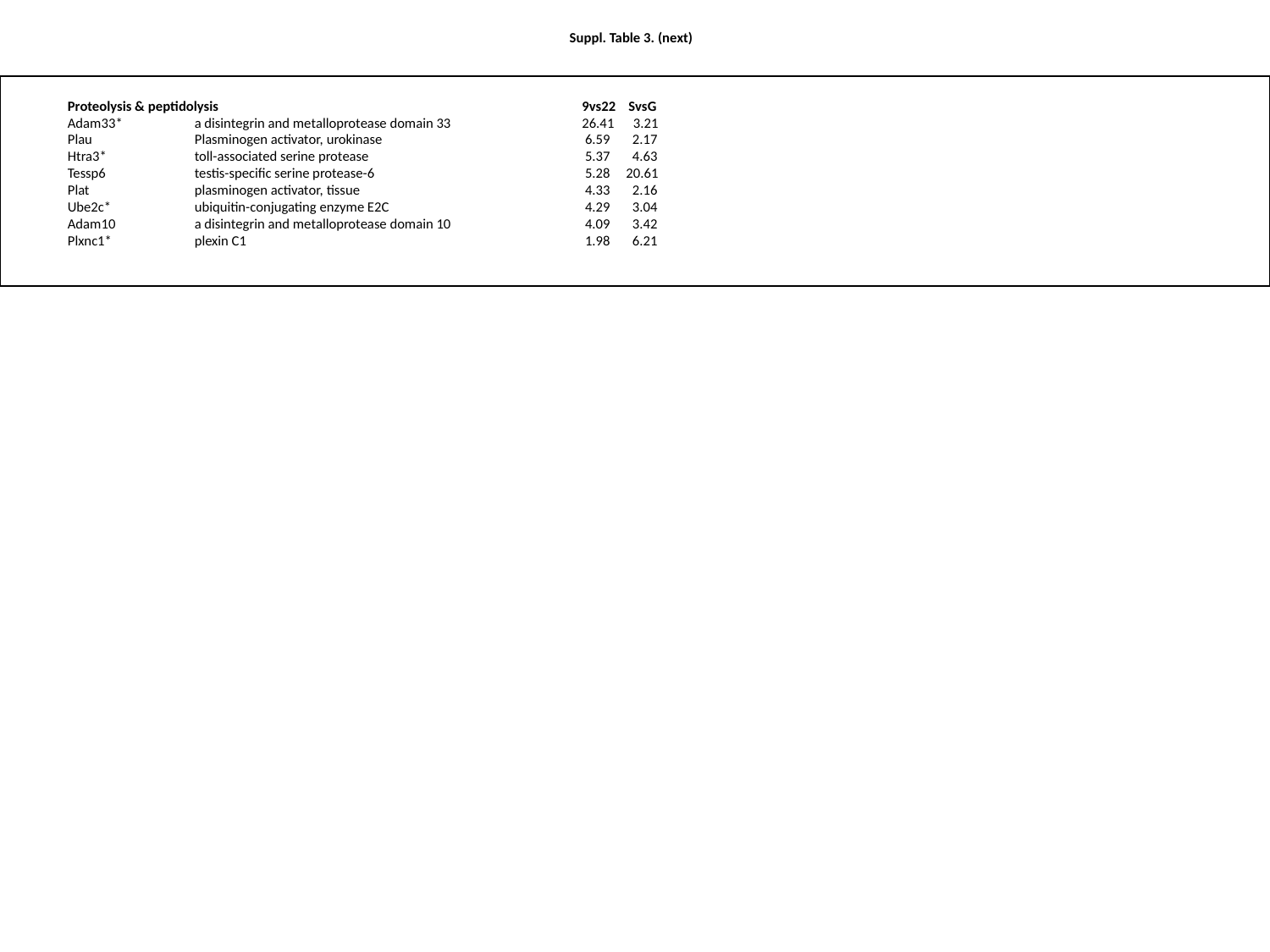

Suppl. Table 3. (next)
Proteolysis & peptidolysis			 9vs22 SvsG
Adam33*	a disintegrin and metalloprotease domain 33 	 26.41 3.21
Plau	Plasminogen activator, urokinase		 6.59 2.17
Htra3*	toll-associated serine protease 		 5.37 4.63
Tessp6	testis-specific serine protease-6		 5.28 20.61
Plat	plasminogen activator, tissue		 4.33 2.16
Ube2c*	ubiquitin-conjugating enzyme E2C		 4.29 3.04
Adam10	a disintegrin and metalloprotease domain 10	 4.09 3.42
Plxnc1*	plexin C1			 1.98 6.21

## Slide 6
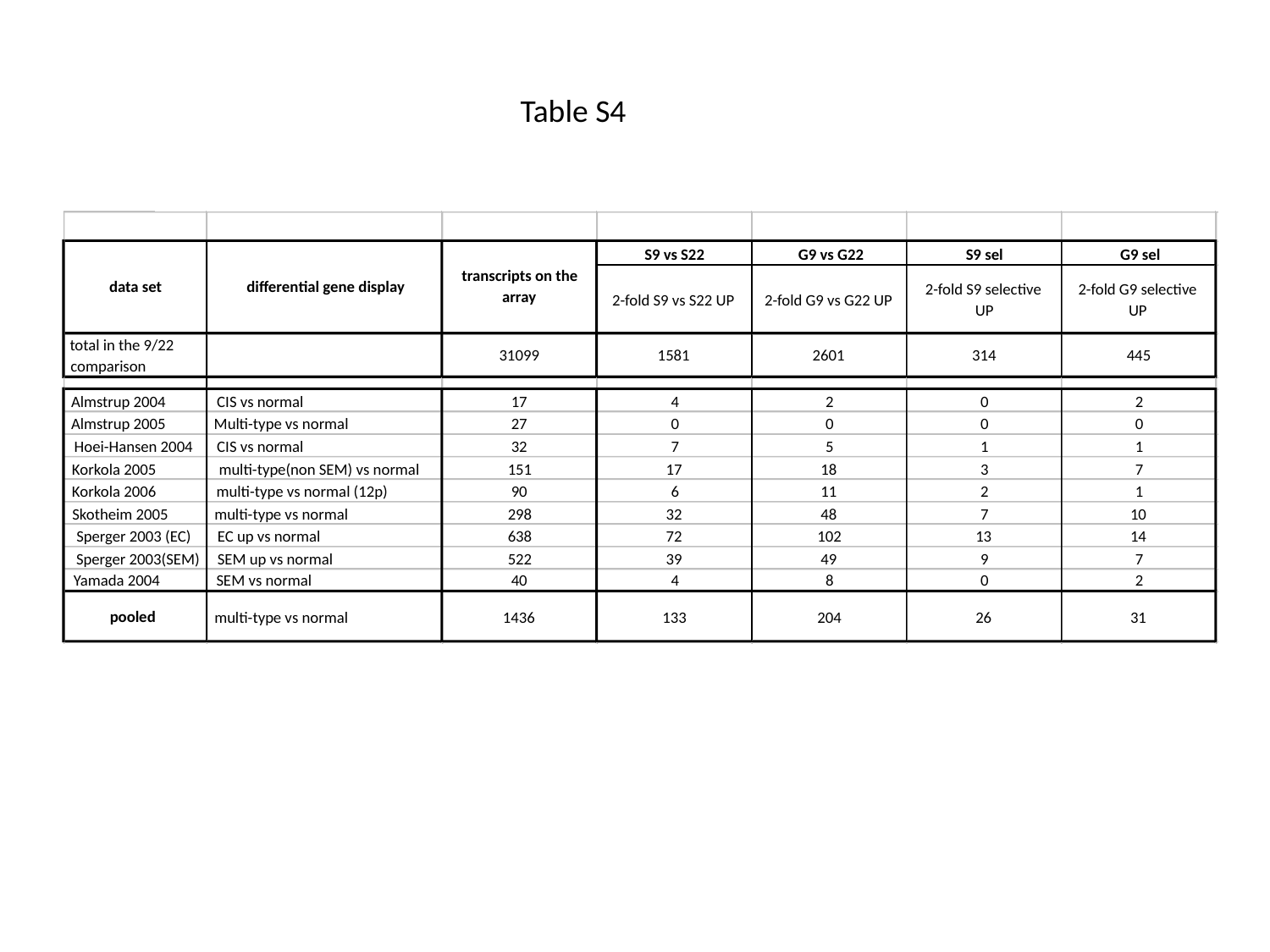

Table S4
S9 vs S22
G9 vs G22
S9 sel
G9 sel
transcripts on the
data set
differential gene display
2-fold S9 selective
2-fold G9 selective
array
2-fold S9 vs S22 UP
2-fold G9 vs G22 UP
UP
UP
total in the 9/22
31099
1581
2601
314
445
comparison
Almstrup 2004
CIS vs normal
17
4
2
0
2
Almstrup 2005
Multi-type vs normal
27
0
0
0
0
Hoei-Hansen 2004
CIS vs normal
32
7
5
1
1
Korkola 2005
multi-type(non SEM) vs normal
151
17
18
3
7
Korkola 2006
multi-type vs normal (12p)
90
6
11
2
1
Skotheim 2005
multi-type vs normal
298
32
48
7
10
Sperger 2003 (EC)
EC up vs normal
638
72
102
13
14
Sperger 2003(SEM)
SEM up vs normal
522
39
49
9
7
Yamada 2004
SEM vs normal
40
4
8
0
2
 pooled
multi-type vs normal
1436
133
204
26
31
