# Supplementary material for: Gene expression profiling of rat spermatogonia and Sertoli cells reveals signaling pathways from stem cells to niche and testicular cancer cells to surrounding stroma
Source: BMC Genomics. 2011 Jan 13;12:29. doi: 10.1186/1471-2164-12-29 (PMC3033334; doi:10.1186/1471-2164-12-29)
Supplement: Additional file 2 — Updated gene lists Description: Tables S1-S4. [file 1471-2164-12-29-S2.DOC]

**Additional file 1**

***Additional files (Figures S1 and S2)***


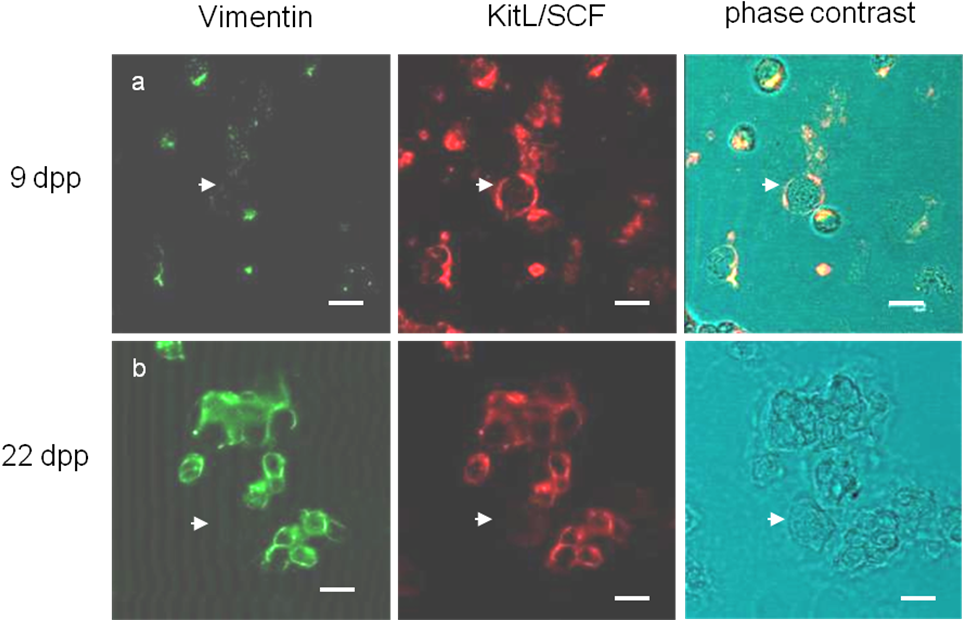


*Figure S1.* KitL/SCF detected in Spga of prepubertal (9 dpp) but not in Spga of pubertal (22 dpp) rats. Immunofluorescence of cells freshly isolated from seminiferous tubules of rat pubs at 9 and 22 dpp, stained with an anti-vimentin (marker for Sertoli cells) or an anti-KitL/SCF antibody. The arrow indicates vimentin-negative Spga.

*
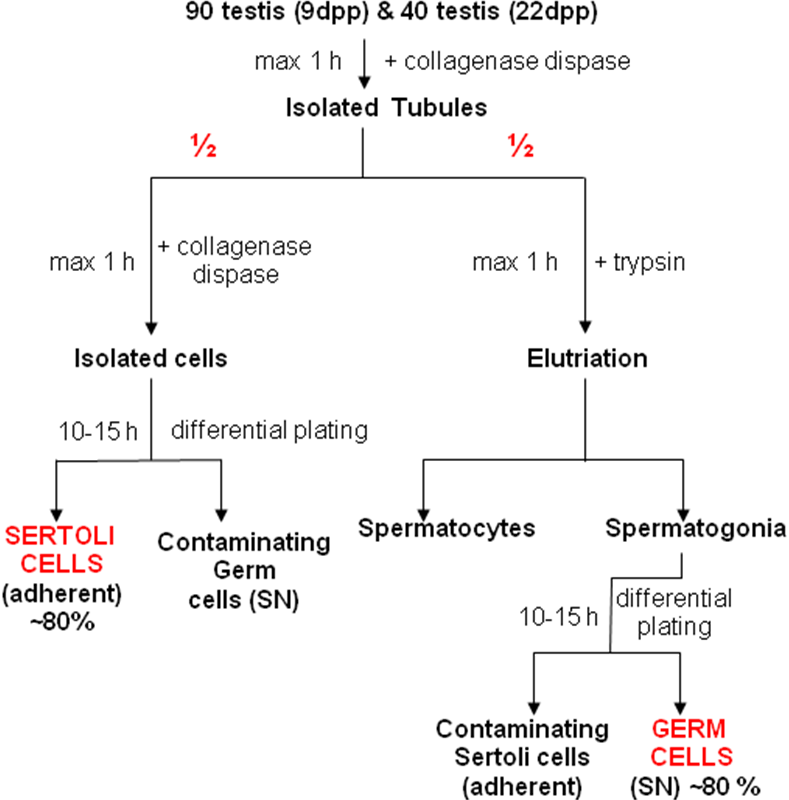
*

*Figure S2.* Schematic over-view of the procedure to purify spermatogonia and Sertoli cells from prepubertal (9dpp) and pubertal (22dpp) rat testis.

***Description of additional files (Tables S1-S4)***

*Table S1:*

In a microarray study, Chamel et al. defined two lists of 1024 transcripts and 230 transcripts corresponding to mitotic and meiotic transcripts, respectively, conserved between rodent male germ cells. We compared these two lists with the different gene clusters defined in Fig 2.

The lists of transcripts enriched in spermatogonia (mitotic, DET MI) and in spermatocytes (meiotic, DET ME) respectively, reported in Chalmel et al. were retrieved from the GermOnline website ([http://www.germonline.org/datasets/Mus_musculus.html](https://oweb/owa/redir.aspx?C=49d2df2352784cbe92c0cbe801c90d59&URL=http%3A%2F%2Fwww.germonline.org%2Fdatasets%2FMus_musculus.html)). Rat orthologs were retrieved thanks to the NetaffxTM Analysis center ([http://www.affymetrix.com/analysis/index.affx](https://oweb/owa/redir.aspx?C=49d2df2352784cbe92c0cbe801c90d59&URL=http%3A%2F%2Fwww.affymetrix.com%2Fanalysis%2Findex.affx)). Only genes for which a probe was present on the rat microarray chip used in the present study were kept for the contingency table analysis.

Number of genes in a cluster which overlap with the 1024 mitotic transcripts and 230 meiotic transcripts from Chamel et al. [21] are given within the rectangles as observed and expected number of genes. The gradual color coded bar shows over-representation (red) and under-representation (blue) of overlapping genes. P-values indicated on the color scale genes were calculated using a Gaussian hypergeometric test (Fischer exact probability test).

*Table S2*

List of the most up-regulated genes in rat spermatogonia cells at 9dpp. The 445 probe sets in the 2-fold selective G9 cluster (shown in figure 2), were classified in 14 molecular and cellular functional groups based on their GO (gene ontology) annotation. Shown are 93 genes represented in 183 probes detecting a minimally 4-fold variation in pair wise comparison 9vs22: mean fold change between Spga at 9dpp (G9) vs 22 dpp (G22). GvsS: mean fold change between Spga at 9dpp (G9) vs Sertoli at 9dpp (S9) (* predicted genes).

*Table S3*

List of the most up-regulated genes in rat Sertoli cells at 9dpp. The 314 probe sets found in the 2-fold selective S9 cluster (shown in figure 2), were classified in 14 molecular and cellular functional groups based on their GO (Gene Ontology) annotation. Shown are 98 genes represented in 172 probes detecting a minimally 4-fold variation in pair wise comparison. 9vs22: mean fold change between Sertoli cells at 9dpp (S9) vs 22 dpp (S22). SvsG: mean fold change between Sertoli cells at 9dpp (S9) vs Spga at 9dpp (G9) (* predicted genes).

*Table S4:*

The lists of transcripts enriched in testis cancers or CIS in comparison to normal tissues or cells were retrieved from the following publications:
K. Almstrup et al., Br J Cancer 92, 1934 (2005) [69] ; K. Almstrup et al., Cancer Res 64, 4736 (2004) [76] ; C. E. Hoei-Hansen et al., Mol Hum Reprod 10, 423 (2004) [70] ; J. E. Korkola et al., Cancer Res 66, 820 (2006) [72] ; J. E. Korkola et al., Oncogene 24, 5101 (2005) [71] ; R. I. Skotheim et al., Cancer Res 65, 5588 (2005) [73] ; J. M. Sperger et al., Proc Natl Acad Sci U S A 100, 13350 (2003) [74] ; S. Yamada et al., DNA Res 11, 335 (2004). [75]. Specific gene lists considered for each study are depicted in supplementary table S4.
Rat orthologs were retrieved thanks to the NetaffxTM Analysis center ([http://www.affymetrix.com/analysis/index.affx](https://oweb/owa/redir.aspx?C=49d2df2352784cbe92c0cbe801c90d59&URL=http%3A%2F%2Fwww.affymetrix.com%2Fanalysis%2Findex.affx)). Only genes for which a probe was present on the rat microarray chip used in the present study were kept for the contingency table analysis. The cluster of genes associated with testis cancer used for figure 5A was a cluster of 1436 transcripts reported at least once to be enriched in tumoral tissue in the aforementioned publications. Fisher’s exact test was used to determine the statistical significance of enrichment/deprivation in the different categories.

**References for the supplement with the reference number in the manuscript:**

21. Chalmel F, Rolland AD, Niederhauser-Wiederkehr C, Chung SS, Demougin P, Gattiker A, Moore J, Patard JJ, Wolgemuth DJ, Jegou B *et al*: The conserved transcriptome in human and rodent male gametogenesis. *Proc Natl Acad Sci U S A* 2007, 104(20):8346-8351.

69. Almstrup K, Hoei-Hansen CE, Wirkner U, Blake J, Schwager C, Ansorge W, Nielsen JE, Skakkebaek NE, Rajpert-De Meyts E, Leffers H: Embryonic stem cell-like features of testicular carcinoma in situ revealed by genome-wide gene expression profiling. *Cancer Res* 2004, 64(14):4736-4743.

70. Hoei-Hansen CE, Nielsen JE, Almstrup K, Hansen MA, Skakkebaek NE, Rajpert-DeMeyts E, Leffers H: Identification of genes differentially expressed in testes containing carcinoma in situ. *Mol Hum Reprod* 2004, 10(6):423-431.

71. Korkola JE, Houldsworth J, Dobrzynski D, Olshen AB, Reuter VE, Bosl GJ, Chaganti RS: Gene expression-based classification of nonseminomatous male germ cell tumors. *Oncogene* 2005, 24(32):5101-5107.

72. Korkola JE, Houldsworth J, Chadalavada RS, Olshen AB, Dobrzynski D, Reuter VE, Bosl GJ, Chaganti RS: Down-regulation of stem cell genes, including those in a 200-kb gene cluster at 12p13.31, is associated with in vivo differentiation of human male germ cell tumors. *Cancer Res* 2006, 66(2):820-827.

73. Skotheim RI, Lind GE, Monni O, Nesland JM, Abeler VM, Fossa SD, Duale N, Brunborg G, Kallioniemi O, Andrews PW *et al*: Differentiation of human embryonal carcinomas in vitro and in vivo reveals expression profiles relevant to normal development. *Cancer Res* 2005, 65(13):5588-5598.

74. Sperger JM, Chen X, Draper JS, Antosiewicz JE, Chon CH, Jones SB, Brooks JD, Andrews PW, Brown PO, Thomson JA: Gene expression patterns in human embryonic stem cells and human pluripotent germ cell tumors. *Proc Natl Acad Sci U S A* 2003, 100(23):13350-13355.

75. Yamada S, Kohu K, Ishii T, Ishidoya S, Hiramatsu M, Kanto S, Fukuzaki A, Adachi Y, Endoh M, Moriya T *et al*: Gene expression profiling identifies a set of transcripts that are up-regulated inhuman testicular seminoma. *DNA Res* 2004, 11(5):335-344.

76. Almstrup K, Ottesen AM, Sonne SB, Hoei-Hansen CE, Leffers H, Rajpert-De Meyts E, Skakkebaek NE: Genomic and gene expression signature of the pre-invasive testicular carcinoma in situ. *Cell Tissue Res* 2005, 322(1):159-165.
